# Automatic Detection Method for Shield Tunnel Segment Dislocation Based on Facility Point Cloud Removal and Segment Segmentation

**DOI:** 10.3390/s26154901

**Published:** 2026-08-03

**Authors:** Kaikun Zhang, Wei Li, Qiuzhao Zhang, Wei Duan, Shubi Zhang, Jian Shi, Wanli Liu

**Affiliations:** 1 School of Environment and Spatial Informatics, China University of Mining and Technology, Xuzhou 221000, China; 2 Nanjing Institute Surveying, Mapping & Geotechnical Investigation Co., Ltd., Nanjing 210019, China; shijian@njcky.com; 3 China Coal Technology and Engineering Group Xi’an Research Institute (Group) Co., Ltd., Xi’an 710077, China; 4 School of Mathematics and Statistics, Xuzhou University of Technology, Xuzhou 221018, China

**Keywords:** shield tunnel, tunnel point cloud filtering, segment segmentation, segment dislocation

## Abstract

**Highlights:**

**What are the main findings?**

- A point cloud filtering method integrating offset features and semantic segmentation was proposed to effectively remove tunnel facility interference while preserving tunnel wall data.
- A tunnel segment segmentation and dislocation detection framework was developed, achieving seam localization and dislocation errors of less than 2 mm.

**What are the implications of the main findings?**

- The proposed method improves the automation, accuracy, and reliability of shield tunnel segment dislocation detection based on mobile laser scanning data.
- The framework provides an efficient solution for tunnel structural health monitoring and supports intelligent operation and maintenance of subway infrastructure.

**Abstract:**

Mobile laser scanning (MLS) has become an effective technique for deformation monitoring in subway shield tunnels. Among various deformation characteristics, segment dislocation is an important indicator of tunnel structural health because it reflects the relative deformation between adjacent segments and may affect the mechanical behavior and waterproof performance of segmental joints. However, existing MLS-based methods for dislocation detection still suffer from facility interference, inaccurate seam localization, and limited automation in quantitative analysis. To address these challenges, this study proposes an automated method for shield tunnel segment dislocation detection based on MLS point cloud processing. The proposed framework consists of three main steps. First, a point cloud filtering strategy integrating offset features and semantic segmentation is developed to remove facility-related noise while preserving tunnel wall information. Second, a tunnel segment segmentation method combining bolt hole extraction and moving template matching is introduced to achieve accurate localization of both horizontal and longitudinal seams, where bolt holes are identified using normal vector and distance constraints. Finally, automated segment dislocation analysis is performed based on the filtering and segmentation results. Experimental results demonstrate that the proposed filtering method improves accuracy by 8.7% and 5.6% compared with conventional ellipse fitting and cylinder fitting methods, respectively. Using manually interpreted reference values derived from the same MLS dataset as the evaluation reference, the proposed method achieves less than 2 mm deviation in both seam localization and dislocation analysis, demonstrating high consistency with manual interpretation. Compared with existing automatic approaches, the proposed method provides more accurate and reliable automated dislocation analysis, significantly reducing the need for manual inspection. The proposed method enhances the automation, consistency, and reliability of shield tunnel deformation assessment and provides an effective solution for structural health monitoring.

## 1. Introduction

With the rapid advancement of urbanization and economic development, rail transit has become a primary solution for alleviating urban traffic congestion due to its safety, convenience, and low environmental impact. As of 31 December 2025, 31 provinces (autonomous regions and municipalities) and the Xinjiang Production and Construction Corps had 54 cities operating urban rail transit systems, comprising 343 lines with a total operating length of 11,710.3 km and 6680 stations [1]. Tunnel sections account for a significant proportion of the total network length. Following the conclusion of the large-scale construction phase, China’s subway development has entered a new stage focused on safe operation and maintenance. Subway tunnels, both during and after construction, are susceptible to various external and internal influences, including nearby construction activities, structural design limitations, operational vibrations, and surrounding soil loads or ground displacements. These factors can lead to serious safety concerns such as deformation, cracking, and water leakage [2,3,4,5]. Consequently, the timely detection of tunnel deformations and defects is essential to ensuring the safe operation of subway systems. However, underground tunnels pose significant challenges for inspection due to poor visibility and limited maintenance windows. Efficient and accurate detection within these constraints remains a major challenge in the field of underground engineering [6].

Currently, tunnel deformation monitoring methods include traditional leveling instruments [7], rangefinders [8], total stations [9], ground-based 3D laser scanners [10], sensor-based systems [11], and mobile laser scanners [12]. Each method has distinct advantages and limitations: while traditional approaches remain applicable in specific scenarios, they are generally inefficient and offer limited spatial coverage, making them unsuitable for modern tunnel engineering requirements; ground-based 3D laser scanners provide high measurement accuracy but face challenges such as large data volumes and the need for registration between different scanning stations; sensor-based methods require the manual deployment of numerous sensors along the tunnel, resulting in low efficiency and high operational costs; mobile 3D laser scanning, with its fast, efficient, and accurate data acquisition capabilities, has emerged as the most effective solution for tunnel deformation monitoring, offering strong technical support for tunnel safety management. The point cloud data collected by mobile systems can be used to invert deformation via 3D coordinate analysis and detect leakage based on reflection intensity, thus enabling comprehensive tunnel condition assessments. However, current techniques still face challenges such as limited detection accuracy, low automation, and poor algorithm adaptability, which constrain their effectiveness in real-world engineering applications.

Based on point cloud data acquired by mobile 3D laser scanners, various types of deformation analyses can be conducted, including cross-sectional convergence analysis, longitudinal settlement monitoring, ellipticity analysis, and segment dislocation detection. Among these, segment dislocation detection is one of the most challenging tasks because it requires accurately quantifying the relative displacement between adjacent segments at segmental joints. Due to the segmented assembly structure of shield tunnel linings, these joints are particularly susceptible to deformation and structural deterioration [13]. Under the effects of uneven geological conditions, ground loss, construction deviations, and long-term operational loads, relative displacement may occur between adjacent segments, resulting in segment dislocation. Excessive dislocation can cause stress redistribution around segmental joints, reduce the load-transfer performance of the lining structure, impair the waterproof performance of tunnel joints, and even induce water leakage or segment cracking. Therefore, segment dislocation is widely regarded as an important indicator for evaluating the deformation behavior and structural health condition of shield tunnels [14,15]. Segment dislocation can be classified into two categories according to its location: intra-ring dislocation, occurring between the upper and lower segments within the same ring (maximum allowable value: ±10 mm), and inter-ring dislocation, occurring between adjacent rings (maximum allowable value: ±15 mm) [16].

For automated dislocation assessment, accurate segment seam positioning is not the final objective but the prerequisite for reliable dislocation quantification. Segment seams define the boundaries between adjacent segments and rings, providing the spatial reference for selecting corresponding point cloud neighborhoods on both sides of each joint. Accurate seam localization therefore enables the relative displacement to be calculated at the correct structural boundaries, allowing intra-ring and inter-ring dislocations to be correctly distinguished, abnormal joints to be spatially localized, and their severity to be quantitatively evaluated for maintenance decision-making. In contrast, seam positioning errors may lead to the comparison of non-corresponding lining surfaces, directly propagating into subsequent dislocation estimation and reducing the reliability of deformation analysis. Therefore, the accuracy of segment seam positioning fundamentally determines the reliability of automated tunnel dislocation detection. Consequently, automated segment dislocation detection requires two key technical challenges to be addressed: (1) accurately and autonomously locating segment seams, and (2) effectively removing interfering noise points.

(1) Segment seam positioning

The lining of a shield tunnel typically consists of six distinct types of segments: one capping segment (designated KP), two adjacent segments (designated BP and CP), and three standard segments (designated A1P, A2P, and A3P). These segments are connected by bolts and assembled according to a predefined pattern [16]. Horizontal seams are formed between segments within the same ring, while longitudinal seams occur between adjacent rings. Existing approaches for seam recognition are mainly categorized into three types: grayscale image-based methods, point cloud geometric feature-based methods, and deep learning-based methods. In grayscale image-based approaches, L. Du et al. [17] detected longitudinal seams using image gradient information, and Sun et al. [18] enhanced Du’s method by incorporating human–computer interaction and azimuth estimation to recognize horizontal seams. In methods based on geometric features, Cheng Yi et al. [19] introduced a segmentation technique using normal vectors and central angles; Chen Lin et al. [20] extracted seam features based on distance differentials; and Lu Jianjun et al. [21] employed bolt hole feature extraction to improve longitudinal seam localization. In deep learning-based approaches, Anbin Yu et al. [22] applied convolutional neural networks (CNNs) to grayscale image-based seam detection; Lu Dening [23] integrated directional neighborhood information into Faster R-CNN to identify bolt holes; Cheng Shu et al. [24] utilized YOLOv3 for orthophoto image analysis; and Zhang et al. [25] proposed a method for generating binary images from unfolded point clouds via tunnel axis extraction, enabling segmentation through image processing techniques. Despite these advancements, current methods still encounter several challenges. The complexity of tunnel structures, presence of noise, and data inhomogeneity can significantly compromise algorithm robustness, while some techniques rely heavily on manual intervention, limiting their level of automation.

(2) Tunnel point cloud filtering

Accurate seam positioning and subsequent deformation analysis rely on the availability of clean tunnel lining point cloud data. However, actual scanning results often contain substantial interference, as in addition to the lining itself, numerous ancillary structures—such as pipelines, contact networks, and rails—are also captured during scanning. In response, various tunnel point cloud filtering methods have been proposed in recent years. For instance, Li Xianshuai et al. [26] combined the least squares method with Lagrange multipliers for ellipse fitting-based denoising; Xiangyang Xu et al. [27] introduced the “circular likelihood” method utilizing tunnel symmetry to eliminate noise; Chen et al. [28] employed a multi-model random sample consensus (RANSAC) algorithm for cross-sectional fitting; and Zhu et al. [29] implemented iterative filtering based on an elliptical cylinder model. More recently, advanced techniques have emerged: Shi Bo et al. [30] proposed a cylindrical projection-based cloth simulation filtering approach to efficiently extract lining point clouds, while Choudhury et al. [31] developed an enhanced bilateral filtering technique that preserves edge features while smoothing, making it suitable for high-precision point cloud processing. In summary, most existing algorithms leverage the structural characteristics of shield tunnels—particularly their circular or elliptical cross-sections—to separate lining point clouds from facility-related noise via ellipse fitting. However, such methods are primarily suited to the early stages of tunnel construction. Once deformation occurs, the cross-sectional shape may deviate from the ideal geometric profile, and rigid ellipse fitting may result in the inadvertent removal of valid lining point data, thereby compromising the accuracy of subsequent analyses.

To address the aforementioned challenges, this paper proposes a point cloud filtering method for shield tunnels that integrates offset features with semantic segmentation, enabling effective separation of facility point clouds from tunnel wall point clouds. Subsequently, a seam positioning algorithm based on bolt hole distribution and a mobile template matching strategy is introduced. By leveraging the spatial distribution of bolt hole point clouds together with prior knowledge of segment assembly patterns, the proposed method improves seam-positioning accuracy in complex environments, thereby providing reliable structural references for extracting corresponding point cloud neighborhoods and quantitatively calculating intra-ring and inter-ring dislocations. The proposed approach was validated using measured point cloud data from an operational subway tunnel. Experimental results demonstrate that the method significantly outperforms existing techniques in both accuracy and efficiency.

## 2. Materials and Methods

**Figure 1:**
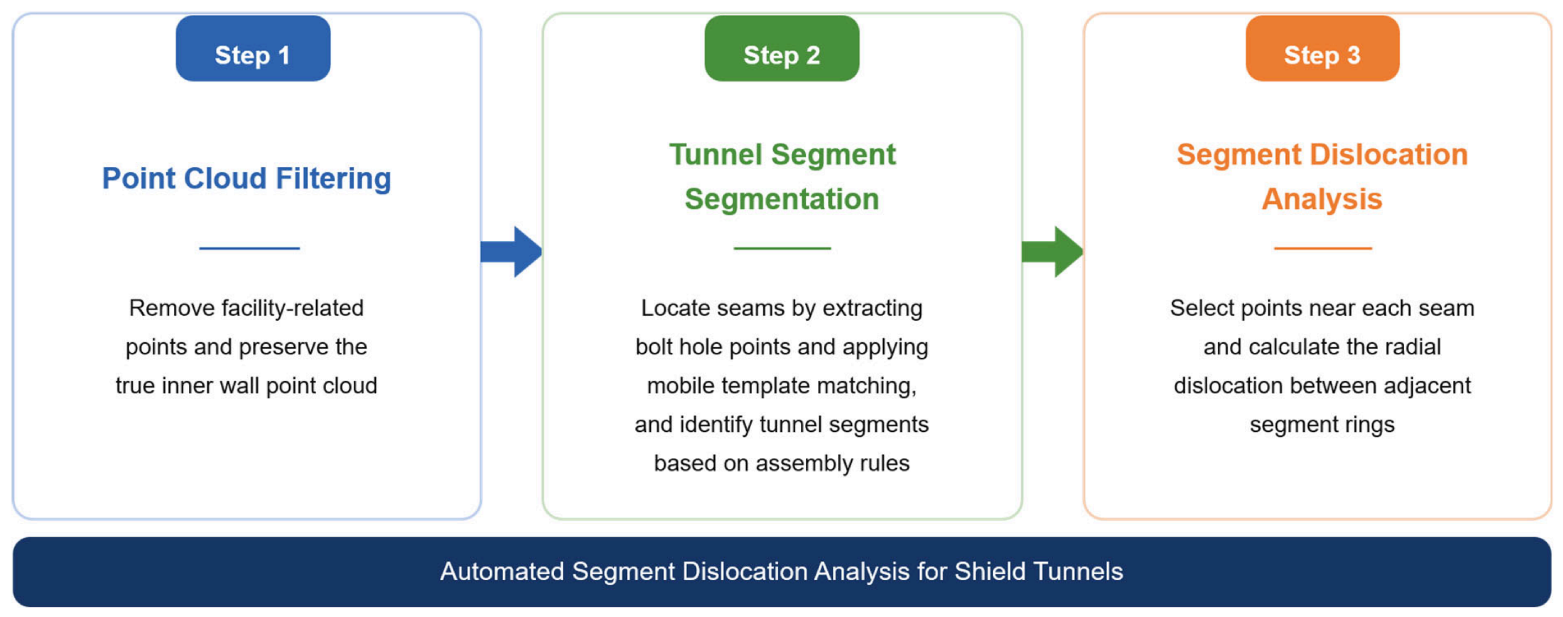
Flowchart of the proposed method.

The proposed method in this paper involves three main steps: removing the facility point cloud within the tunnel, segmenting the tunnel lining by locating the seams, and subsequently analyzing segment dislocation based on the processed data. First, a point cloud filtering method for shield tunnels is introduced, which combines offset features with semantic segmentation to effectively eliminate facility-related noise in complex environments while preserving the true inner wall point cloud. Building upon this, a tunnel segment segmentation approach is proposed that integrates bolt hole point cloud extraction with a mobile template matching strategy. By leveraging the spatial distribution of bolt holes and established assembly rules, the positioning of horizontal seams is transformed into an optimal matching problem between bolt holes and the moving template, while longitudinal seams are fitted segment by segment. Finally, the dislocation of tunnel segments on an operational subway line is automatically analyzed using the results of the filtering and segmentation process. The overall workflow of the proposed method is illustrated in Figure 1.

### 2.1. Point Cloud Filtering Method for Subway Shield Tunnels Integrating Offset Features and Semantic Segmentation

**Figure 2:**
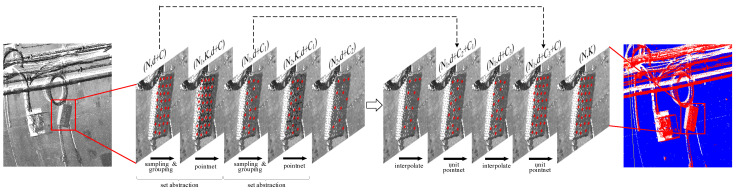
PointNet++ segmentation branch network structure.

To mitigate the interference of noise point clouds while preserving relevant tunnel structure information, this paper incorporates deep learning techniques, leveraging their robust feature-learning capabilities to more accurately identify noise points and facility-related point clouds. To enhance the local geometric representation capability of the semantic segmentation network, an offset feature extraction module is introduced, coupled with local translation and normalization operations to minimize data distribution discrepancies. This approach significantly improves the model’s adaptability in complex tunnel environments. As a result, it effectively retains the true tunnel inner wall point cloud while removing noise, thereby providing high-quality data support for tunnel monitoring and detection tasks. The semantic segmentation network employed in this study is based on the classic PointNet++ architecture [32], as shown in Figure 2.

Point cloud semantic segmentation methods based on deep learning typically utilize local geometric features or global semantic features for category classification. However, in shield tunnel environments, the local geometric distributions of tunnel walls and facility point clouds are highly similar, presenting several challenges for traditional methods: (1) Both the tunnel wall and facility point clouds reside within a confined space resembling a cylinder, with local features such as surface curvature and normal vectors being similar in boundary areas, making it difficult for the network to differentiate them based on geometric features. (2) The tunnel wall and facilities are strongly coupled in the global structure (e.g., cables extending along the tunnel wall and pipelines running parallel), creating similar spatial topologies in local neighborhoods and exacerbating feature confusion. Relying solely on long-range dependency modeling may lead to mis-segmentation due to excessive global correlation. (3) The distribution of tunnel facilities (e.g., pipelines and rails) is influenced by construction errors and maintenance adjustments, lacking regularity, and making it challenging to achieve robust segmentation through prior distribution modeling.

To address the aforementioned challenges, this paper identifies that the prior structure of the shield tunnel cross-section, which approximates a circle, contains critical separability information. Specifically, the distance from the facility point cloud to the center of the cross-section is much smaller than that of the tunnel wall point cloud. This feature is closely linked to the semantic category of the point cloud but has not been explicitly leveraged in traditional feature extraction methods. To exploit this, an offset feature extraction module is proposed, which computes the normalized distance from the point cloud to the tunnel center and creates a structured offset feature with category discrimination. This approach effectively utilizes the geometric information of the tunnel structure, thereby enhancing the robustness of the deep learning network. The specific steps are as follows:

First, extract the point cloud set in the form of a cross-section and apply a fitting procedure to obtain the radius and center of the circle by minimizing the objective function F using the random sampling consistency algorithm. Next, calculate the radial distance of each point to the center of the cross-section and the difference between this distance and the radius of the fitted circle. The formula for this calculation is as follows:

(1) $$F=\sum_{i=1}^{n}{\left(\sqrt{(x_{i}-x_{0}{)}^{2}+(z_{i}-z_{o}{)}^{2}}-r_{0}\right)}^{2}$$

(2) $$D_{i}=\sqrt{(x_{i}-x_{o}{)}^{2}+(z_{i}-z_{o}{)}^{2}}$$

(3) $$\Delta D_{i}=D_{i}-r_{0}$$

Among them, $\left(x_{i},z_{i}\right)$ represents the coordinates of the ith point in the point cloud, n is the total number of point clouds, $r_{0}$ is the radius of the cross-section point cloud fitting, and $\left(x_{0},y_{0},z_{0}\right)$ is the center of the fitted circle.

Tunnel point cloud data is often influenced by various factors, including tunnel structure, environmental conditions, and mileage variations, leading to significant differences in the X, Y, and Z directions. To mitigate these scale discrepancies and ensure the stability and consistency of the neural network when processing diverse tunnel data, this paper applies local translation and normalization to the point cloud data in each segment.

**Figure 3:**
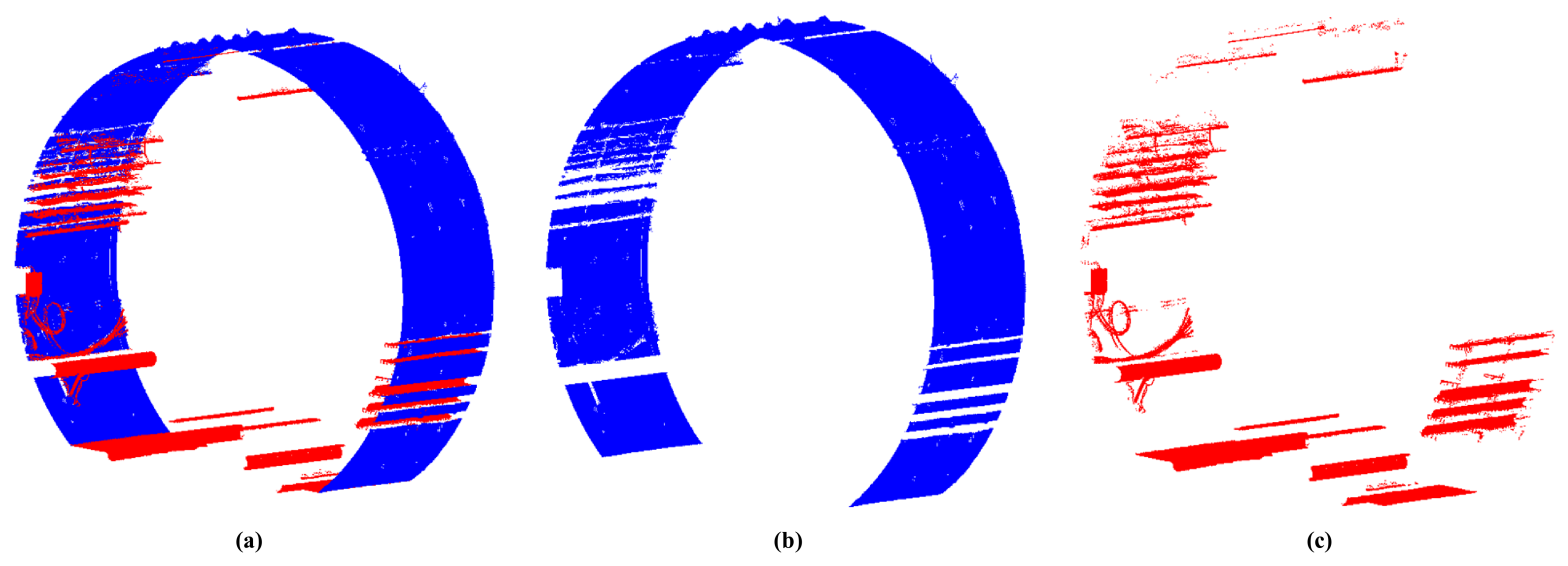
Schematic diagram of the filtering method in this paper. (**a**) Tunnel point cloud semantic segmentation; (**b**) tunnel wall point cloud; (**c**) noise point cloud.

Finally, the original point cloud information is concatenated with offset features, local translation features, and normalized features to generate 7-dimensional point cloud data. These enhanced point clouds are then fed into the PointNet++ semantic segmentation network for training and prediction, allowing for accurate separation of the tunnel wall and facility point clouds based on the predicted labels, as shown in Figure 3.

### 2.2. Shield Tunnel Segment Segmentation Method Based on Bolt Hole Point Cloud Extraction and Moving Template

The segment segmentation algorithm proposed in this paper is based on two key observations: (1) bolt holes manifest as local depressions in the point cloud, which can serve as positioning markers for segment segmentation; (2) segment types and assembly patterns adhere to the rules of prefabricated templates, allowing dynamic template matching to accommodate installation deviations in actual point clouds. The main steps involve bolt hole point cloud extraction and mobile template matching.

#### 2.2.1. Bolt Hole Point Cloud Extraction

(1) Point cloud expansion

**Figure 4:**
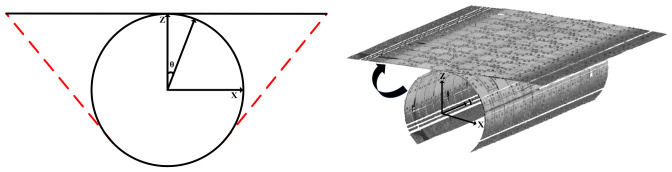
Schematic diagram of point cloud expansion based on cylindrical coordinate system.

The distribution of shield tunnel point clouds in three-dimensional space typically forms a cylindrical structure. The curvature of this structure complicates the spatial differences between the bolt hole and the tunnel inner wall point clouds, making it difficult to quantify these differences effectively using traditional geometric analysis methods. To emphasize the local concave features of the bolt hole and address the spatial discrepancy between it and the tunnel inner wall point cloud, this paper employs a point cloud expansion method based on a cylindrical coordinate system to map the tunnel point cloud to an approximate plane coordinate system. Suppose there is a point in the tunnel with coordinates before expansion of $P_{1}(x_{1},y_{1},z_{1})$, coordinates after expansion of $P_{2}(x_{2},y_{2},z_{2})$, tunnel design radius of R, point N as a point on the positive direction of the axis, and $O(x_{0},y_{0},z_{0})$ as the cross-section fitting center. $\alpha_{c}$ is $∠NOP_{1}$ in radians. Then the coordinates after expansion can be calculated by the following formula. The schematic diagram of the point cloud expansion based on the cylindrical coordinate system is shown in Figure 4.

(4) $$\{\begin{array}{c} x_{2}=\{\begin{array}{c} a_{c}\cdot R\;\;\;\;\;\;\;x_{1}>x_{0}\\ {-a}_{c}\cdot R\;\;\;\;\;\;\;x_{1}<x_{0} \end{array}\\ y_{2}=y_{1}\\ z_{2}=\sqrt{{\left(x_{1}-x_{0}\right)}^{2}+{\left(z_{1}-z_{0}\right)}^{2}} \end{array}$$

(2) Bolt hole point cloud extraction method based on normal vector–distance dual constraints

**Figure 5:**
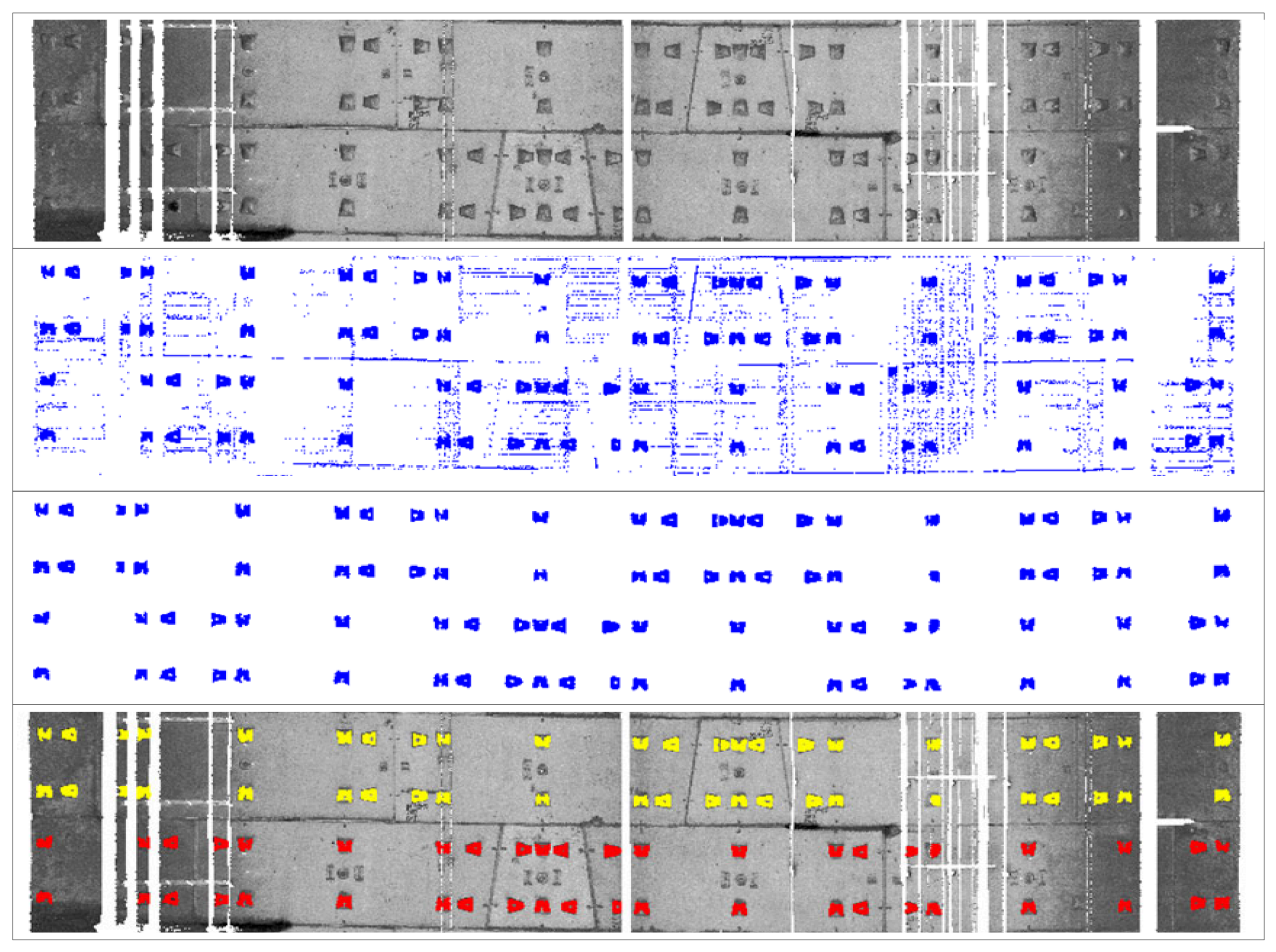
Schematic diagram of bolt hole point cloud extraction based on normal vector and distance dual constraints.

After the point cloud expansion process, the bolt hole point cloud information can be effectively extracted, as shown in Figure 5. The expanded point cloud structure makes the features of both the tunnel wall and bolt hole more distinct, facilitating accurate extraction. Specifically, the normal vector of the tunnel wall point cloud exhibits strong uniformity, typically showing a consistent distribution in the local area, while the normal vector of the bolt hole point cloud displays considerable irregularity and discreteness. This discrepancy in normal vectors becomes a key feature for distinguishing bolt hole point clouds from tunnel wall point clouds. Furthermore, the spatial distribution of bolt holes exhibits clear patterns: they are generally located on the same side of the tunnel wall and follow certain regularities in their longitudinal or horizontal positioning. By combining the distance information of each point in the point cloud to the fitted tunnel wall, the spatial distribution characteristics of the bolt hole point cloud can be further explored.

The main steps are as follows: First, the expanded point cloud is divided into smaller segments of 1.2 m × 1.2 m for subsequent processing. The bolt hole point cloud is extracted by applying dual constraints based on the normal vector and distance for each segment. The plane equation of the segment’s point cloud is fitted using the RANSAC algorithm to calculate the angle $NP_{i}$ between the normal vector $n_{i}$ of each point (the normal vector of a point is obtained by fitting the plane normal vector within its local neighborhood) and the plane’s normal vector $n_{p}$, as well as the distance $d_{i}$ of each point from the fitted plane. By setting angle and distance thresholds, it is determined whether the point cloud belongs to the bolt hole. Next, density-based spatial clustering of applications with noise (DBSCAN) is applied to cluster the extracted bolt-hole point cloud. By adjusting the radius and minimum sample parameters, noise points within the bolt hole point cloud are removed. Finally, the bolt hole point cloud is clustered based on the distance between the bolt holes, and the bolt holes within the same ring are separated.

(5) $$\{\begin{array}{c} NP_{i}=\arccos(\frac{n_{i}\cdot n_{p}}{\left\|n_{i}\right\|\cdot \left\|n_{p}\right\|})\\ d_{i}=\frac{\left|ax_{i}+by_{i}+cz_{i}+d\right|}{\sqrt{a^{2}+b^{2}+c^{2}}} \end{array}$$

#### 2.2.2. Segment Segmentation Method Based on Bolt Hole Point Cloud

**Figure 6:**
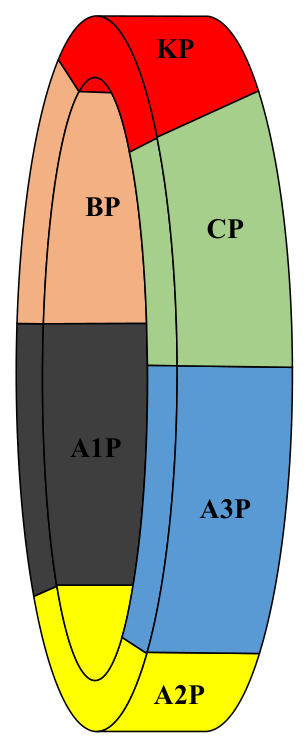
Schematic diagram of single ring segment assembly.

**Figure 7:**
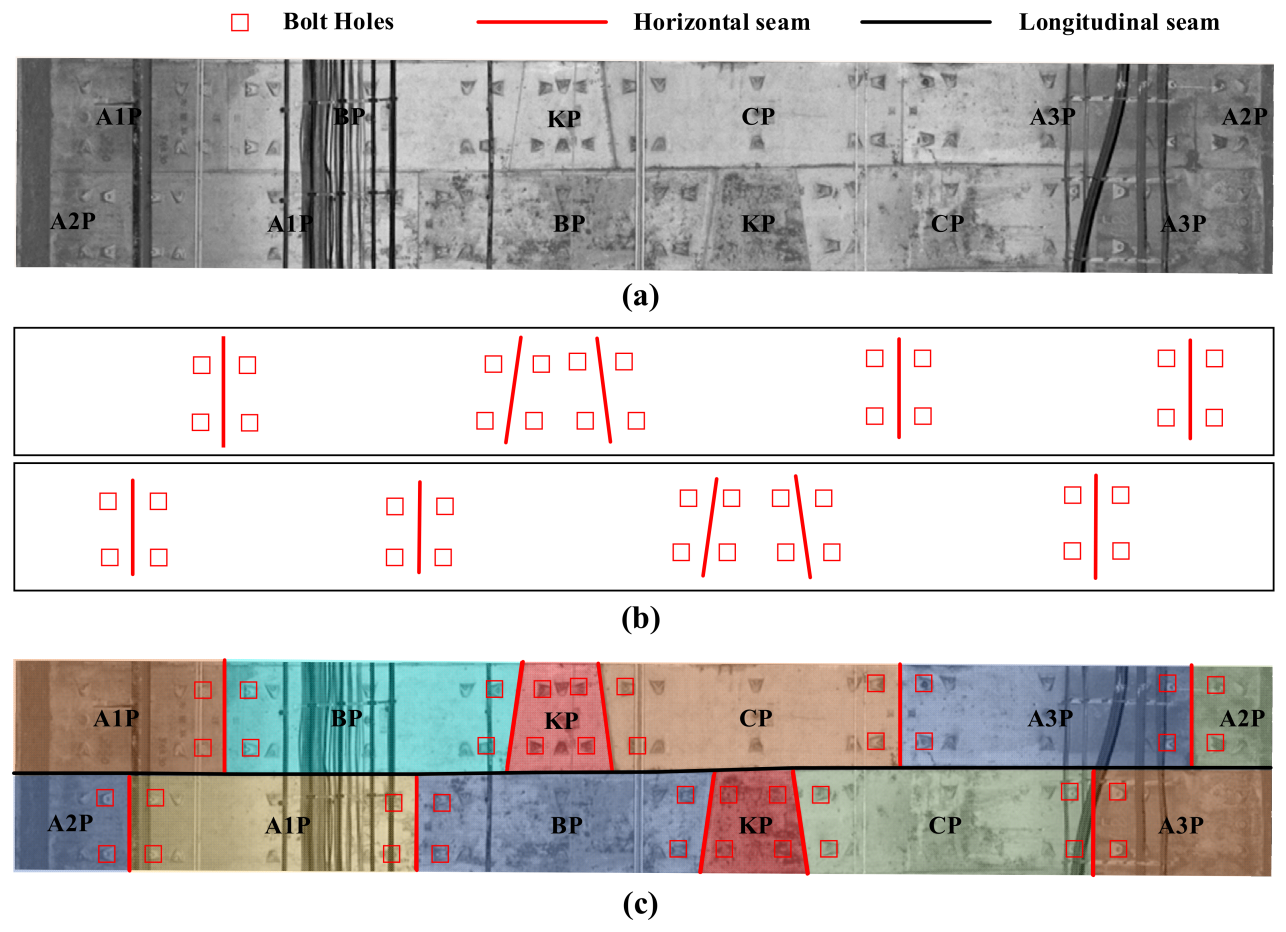
Schematic diagram of the segment segmentation principle based on moving template matching. (**a**) Segment; (**b**) template; (**c**) segmentation result.

The lining of a shield tunnel is typically assembled from six different types of segments, connected by a specific number of bolts and arranged in a fixed pattern. The schematic diagram of the single-ring segment assembly is shown in Figure 6. Based on the distribution patterns of the segments and the characteristics of the horizontal seams located between the bolt holes, this paper proposes a seam positioning method using a moving template. The principle of segment segmentation based on moving template matching is illustrated in Figure 7. The method begins by employing the moving template energy function to roughly locate the horizontal seams, determining their approximate positions by calculating the matching degree with the standard template. Next, based on the initial positioning of the horizontal seams, the bolt hole locations are used to precisely locate the longitudinal seams, with their exact positions determined by analyzing the arrangement and distribution of the bolt holes. Finally, the point cloud data are segmented according to the seam positions to obtain point cloud data for the individual segments, which are then further segmented and fitted to accurately locate the longitudinal seams.

**Figure 8:**
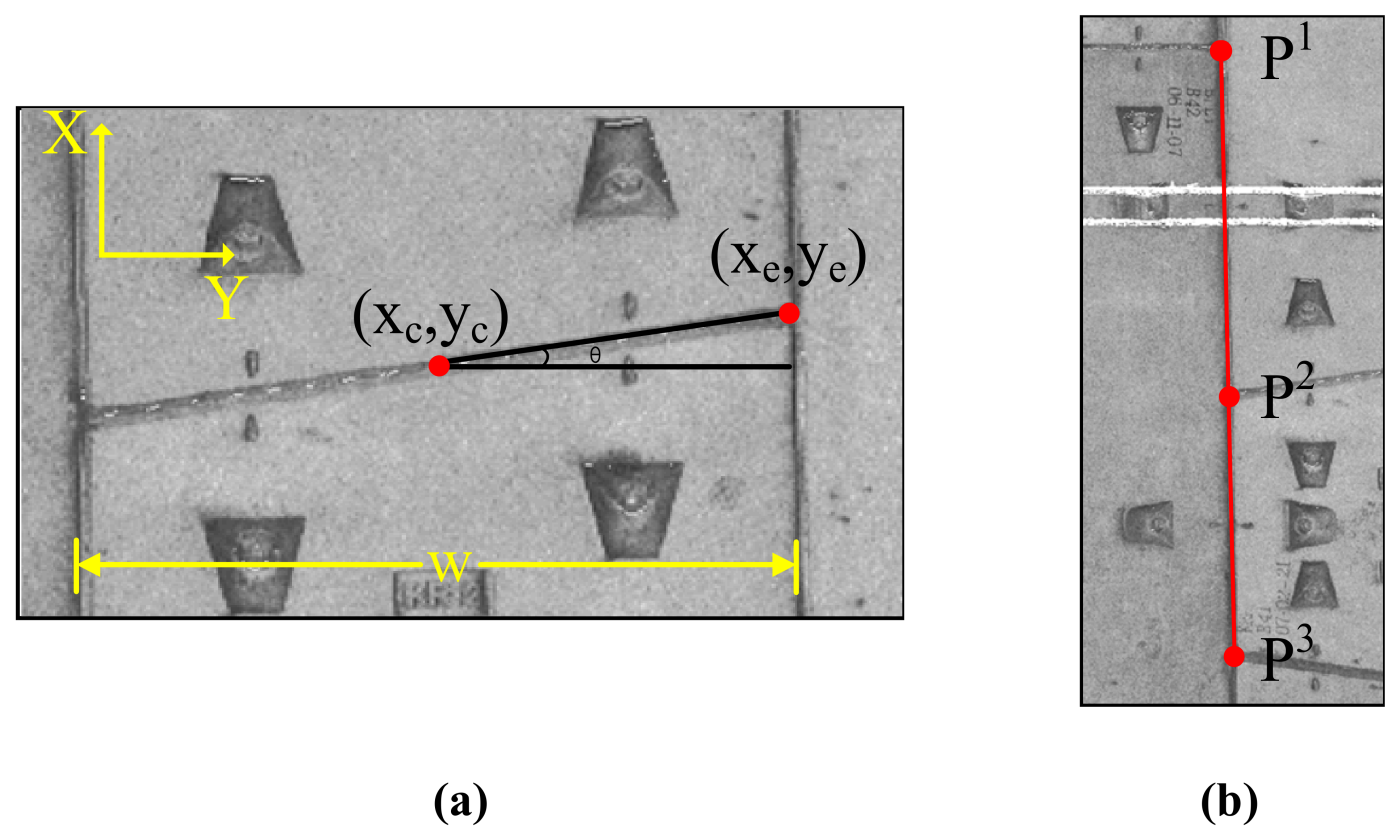
Schematic diagram of segmented fitting of the longitudinal seam. (**a**) Schematic diagram of feature endpoint determination for tunnel segments; (**b**) segmented fitting result of the longitudinal seam.

(1) Horizontal seam positioning method based on mobile template
  (a) Coarse positioning
    The arrangement pattern of the segments in the tunnel can effectively help identify the approximate location of the horizontal seams. This paper redefines the horizontal seam positioning problem as an optimal matching problem between the bolt hole point cloud and a moving template. First, a template is created based on the arrangement pattern of the tunnel segments. The template is then moved and traversed across the bolt hole point clouds of each ring to find the best matching position. The template consists of several position points (e.g., a, b, c, d, e). For each position point, the difference in distance to the center of the nearest bolt hole point cloud above and below is calculated. The position with the smallest sum of these distance differences is selected as the rough position of the horizontal seam. To ensure the correct positioning, an error matching penalty mechanism and an invalid point elimination mechanism are introduced. When the actual distance between the template position and the point cloud is too close or too far (i.e., clearly incorrect), the total distance difference is set to a higher value, effectively excluding the incorrect position. Additionally, if the sum of the distances between the template and the nearest bolt hole point clouds above and below exceeds a certain threshold, it suggests that some bolt hole point clouds may be missing. In such cases, the position point is marked as invalid, and the final optimal position is determined using other valid points from the template.
  (b) Precision positioning
    In tunnel construction, although the assembly of pipe segments follows specific guidelines, actual construction processes often lead to inconsistencies in the size of the gaps between segments. These uneven gaps make it difficult for the rough positioning method to accurately determine the position of the horizontal seam. Therefore, it is necessary to refine the rough positioning by using the bolt hole point cloud. In this case, the position of the horizontal seam is determined by the rough positioning and the center coordinates of the upper and lower bolt hole point clouds. If the upper or lower bolt hole point clouds at the rough positioning location are missing, the positioning approach for this point will follow the same method as the rough positioning.
(2) Longitudinal seam positioning method based on segmented fitting
  In shield tunnel construction, the segments are typically assembled from six different types. However, due to construction errors and complex geological conditions, the longitudinal seams between adjacent rings often do not align perfectly, resulting in significant deviations from the design values. To improve the accuracy of longitudinal seam positioning, this paper proposes a method based on segment fitting. The principle behind this method is as follows: after accurately locating the horizontal seam, the six segments can be identified. By utilizing the seam positions and segment widths, the endpoints where the segments connect can be determined. These endpoints, when connected in sequence, form a segmented fitting broken line that represents the longitudinal seam. The schematic diagram of the segmented fitting of the longitudinal seam is shown in Figure 8. The coordinates of these endpoints can be determined using the following formula:
  (6) $$\{\begin{array}{c} x_{e}=x_{c}+\frac{w}{2\cos(\theta )}\sin(\theta )\\ y_{e}=y_{c}+\frac{w}{2\cos(\theta )}\cos(\theta ) \end{array}$$
  Among them, $\left(x_{c},y_{c}\right)$ is the coordinate of the center point of the horizontal joint, $\left(x_{e},y_{e}\right)$ is the coordinate of the extension point, θ is the angle between the horizontal seam and the y axis, and w is the width of the segment.

### 2.3. Segment Dislocation Analysis

Regarding the calculation of dislocation, this study defines dislocation as the radial offset between the surfaces of adjacent segment rings across a tunnel joint. Taking the inter-ring dislocation as an example, the longitudinal joint is first located. Then, point clouds within 10 cm on both sides of the joint are extracted as the dislocation measurement points for the two adjacent segment rings. The radial distance from each measurement point to the center of the same fixed reference cross-section is calculated, and the difference between the two radial distances is defined as the inter-ring dislocation. The fixed reference cross-section center is defined as the midpoint of the line connecting the fitted cross-section centers of the two adjacent segment rings.

Unlike traditional methods, which calculate the radial distance from each measurement point to the center of its corresponding fitted cross-section, the method proposed in this paper uses a fixed reference cross-section center shared by both sides of the joint. This is because the fitted cross-section centers of adjacent segment rings may not coincide in the horizontal and vertical directions. When an entire segment ring undergoes rigid-body translation, its fitted cross-section center may move together with the segment ring, resulting in little or no change in the relative distance between the measurement point and its corresponding cross-section center. Therefore, the actual radial displacement between adjacent segment rings may be underestimated.

## 3. Results

To evaluate the effectiveness of the proposed algorithm, experiments were conducted using field-acquired point cloud data collected from a shield tunnel section of the Nanjing Metro line. The total scanned tunnel length was approximately 40 km. The test section is a circular shield tunnel with a design radius of 2.75 m and contains numerous pipelines and other ancillary facilities. The data were collected using the NJCK-Track One mobile tunnel inspection system developed by Nanjing Institute of Surveying, Mapping & Geotechnical Investigation, Co., Ltd. The system integrates laser scanners, color line-scan cameras, odometers, and other sensing modules.

The laser scanning module equipped in the system has a maximum scanning rate of 2.18 million points per second, with an adjustable rotational speed ranging from 50 Hz to 200 Hz and a ranging accuracy better than 1.2 mm + 10 ppm. The inspection vehicle supports automatic variable-speed operation from 0.9 to 4.5 km/h, enabling efficient acquisition of high-density point cloud data in long underground tunnel environments. In addition, the system is equipped with nine 8K color line-scan cameras, achieving an imaging resolution of 0.2 mm, which can support the detection of tunnel clearance, deformation, cracks, water leakage, and surface spalling. The acquired high-resolution point cloud data provide reliable data support for tunnel segment localization and dislocation analysis.

### 3.1. Comparison of Filtering Effects

To evaluate the performance of the filtering method proposed in this study, a dataset was constructed from measured tunnel point-cloud data and divided into training and test sets at a ratio of 4:1. The network models were trained and tested on a workstation equipped with an AMD EPYC 9654 processor using 16 CPU cores and an NVIDIA GeForce RTX 4090 GPU with 24 GB of memory. The experiments were conducted on Ubuntu 20.04, and the network models were implemented in PyTorch 1.11.0 using Python 3.8 and CUDA 11.3 for GPU acceleration. The models were trained for 128 epochs using the Adam optimizer, with a batch size of 32, an initial learning rate of 0.001, and a learning-rate decay factor of 0.7.

**Figure 9:**
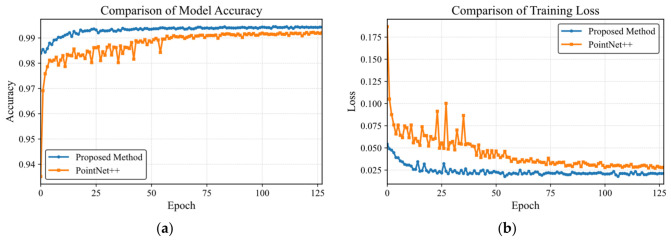
Comparison of training accuracy and loss. (**a**) Training accuracy curve of the model; (**b**) training loss curve of the model.

As shown in Figure 9, the proposed method incorporating offset features achieved marked improvements in semantic segmentation performance compared with the baseline PointNet++ model. During training, the enhanced model converged faster, attained higher segmentation accuracy, and reached a lower final loss. These results indicate that incorporating offset features improves both segmentation accuracy and training efficiency.

The observed improvement can be attributed to the introduction of the circular cross-sectional prior of the shield tunnel into the semantic segmentation process through the offset feature. Conventional point cloud segmentation methods mainly classify points according to their coordinates, normal vectors, and local neighbourhood geometry. These features are generally sufficient when ancillary facilities are clearly separated from the tunnel lining. However, when cables, brackets, or pipelines are closely attached to the tunnel wall, facility points and lining points may exhibit similar Euclidean distances, local densities, and neighbourhood structures, making them difficult to distinguish using local geometric features alone. The offset feature characterizes the deviation of each point from a reference contour derived from the circular tunnel cross-section. Lining points generally exhibit relatively small offsets, whereas facilities protruding from the tunnel wall tend to produce different offset responses, even when they are spatially close to the lining. The offset feature therefore provides a global structural constraint that complements the local features learned by PointNet++, improving the separability of closely adjacent lining and facility points. In this way, the proposed method specifically addresses the misclassification that occurs when ancillary facilities are densely distributed near or directly attached to the tunnel wall.

**Figure 10:**
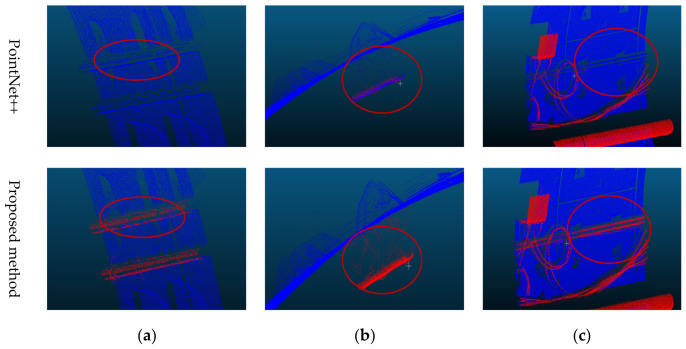
Comparison of segmentation results between this method and PointNet++. (**a**) Sloped-upward pipe; (**b**) upward-sloped pipeline; (**c**) left-side cable.

The comparison of the prediction results obtained by the proposed method and PointNet++ on the test set is presented in Figure 10.

Most existing semantic segmentation algorithms do not explicitly incorporate tunnel cross-sectional priors and may therefore exhibit limited adaptability to complex and variable facility arrangements. To evaluate the robustness of the proposed method under such conditions, shield tunnel point clouds containing different facility layouts were selected for testing. As shown in Figure 10, PointNet++ produced visible confusion between the tunnel lining and nearby ancillary facilities, particularly when pipelines or cables were closely attached to the lining surface. By contrast, the proposed method more clearly delineated the boundaries between the two classes and retained a more complete tunnel wall point cloud while removing facility points. These qualitative results are consistent with the performance improvement shown in Figure 9 and illustrate the practical role of the offset feature in separating spatially adjacent points with similar local geometric characteristics. The resulting filtered point cloud provides a more reliable input for subsequent structural partitioning, deformation monitoring, and disease localization.

The offset feature extraction module is not restricted to PointNet++ and can, in principle, be incorporated into other point cloud semantic segmentation networks. PointNet++ was used in this study as the baseline architecture to evaluate its effectiveness in shield tunnel point cloud filtering.

To further compare the filtering performance of the proposed method with other commonly used techniques, this study employed the open-source software CloudCompare to manually separate non-tunnel wall point clouds as ground truth for filtering evaluation. Three standard metrics—accuracy, precision, and recall—were used to quantitatively assess and compare the filtering effectiveness of different methods.

**Figure 11:**
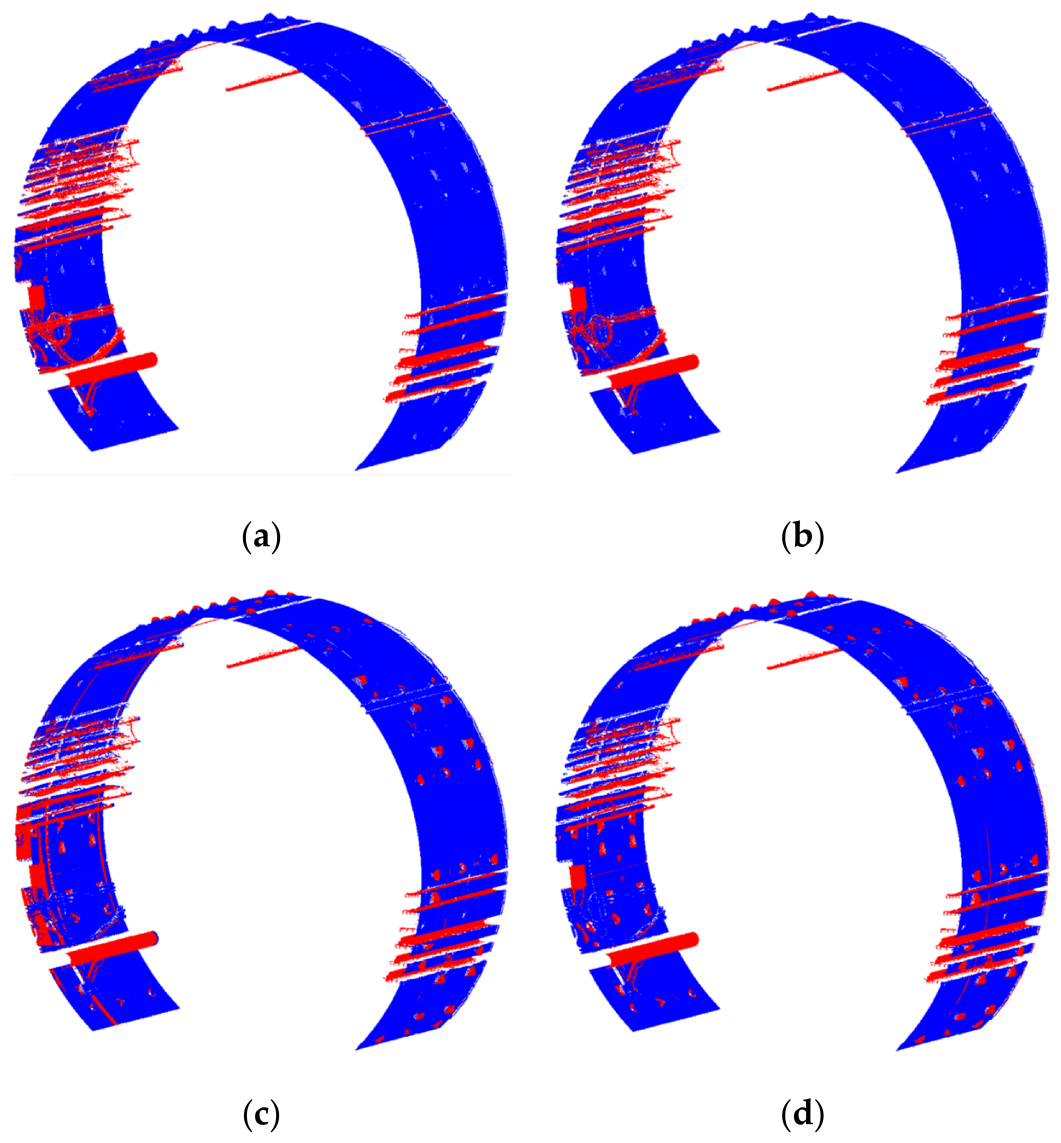
Comparison of the results of this method with those of ellipse fitting filtering and cylinder fitting filtering. (**a**) Truth value; (**b**) proposed method; (**c**) ellipse fitting filtering; (**d**) cylindrical fitting filtering.

**Figure 12:**
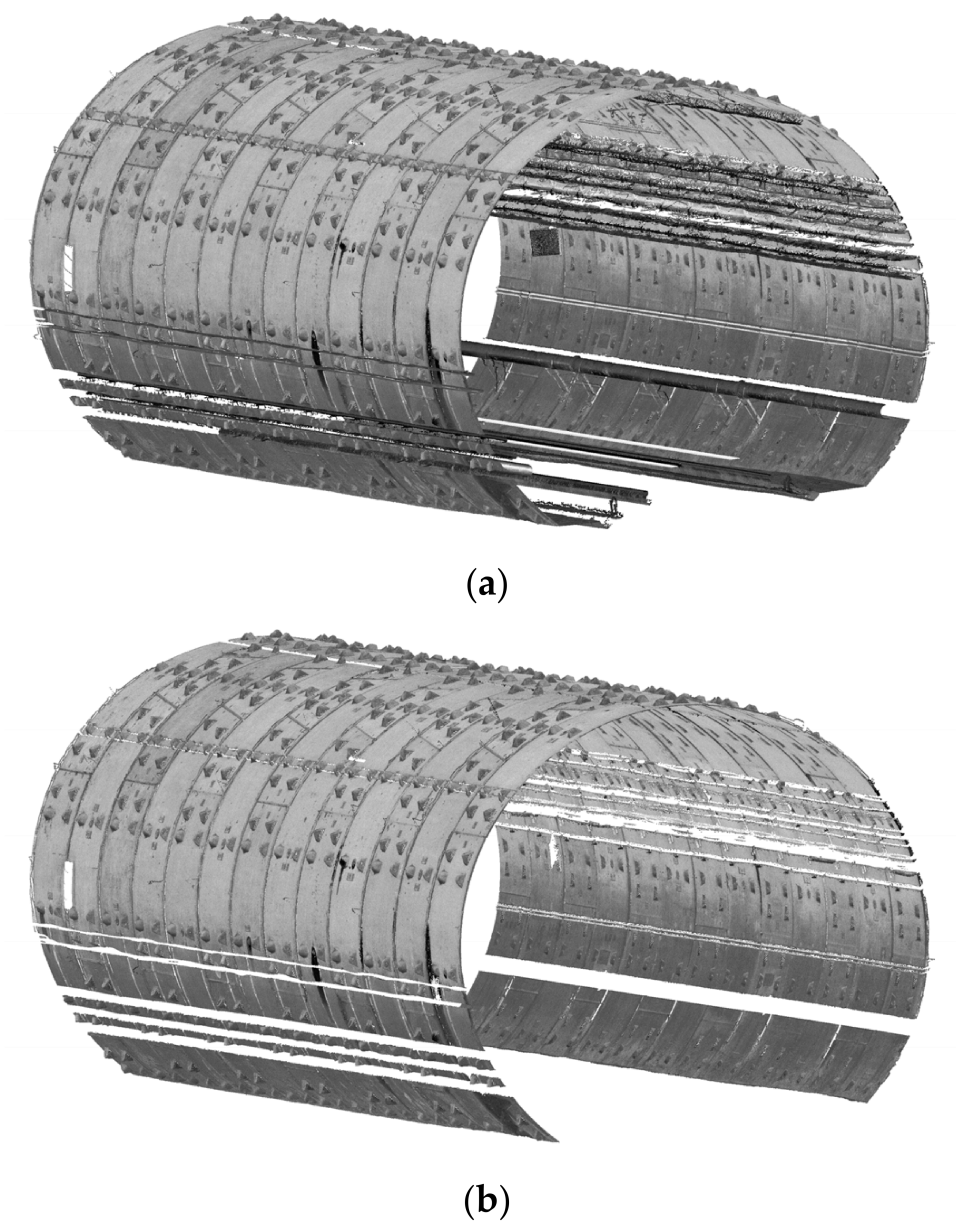
Comparison of results before and after filtering by the proposed method. (**a**) Tunnel point cloud before filtering; (**b**) tunnel point cloud after filtering.

According to the experimental results presented in Figure 11 and Table 1, the filtering method proposed in this paper significantly outperforms the two comparison methods—ellipse fitting and cylinder fitting—across multiple key evaluation metrics, demonstrating superior accuracy and reliability. Specifically, the proposed method achieves a filtering accuracy that is 8.7% and 5.6% higher than that of the ellipse fitting and cylinder fitting methods, respectively. Although the overall shape of the tunnel wall approximates an ellipse, in practice it deviates from an ideal geometric form due to influences such as construction techniques, geological conditions, and settlement-induced deformations. These factors can lead to local irregularities or distortions. Consequently, methods based solely on ideal geometric assumptions (e.g., ellipse or cylinder fitting) exhibit clear limitations: they may fail to remove all noise points effectively, thereby compromising filtering precision, or erroneously eliminate valid tunnel wall point clouds, thus reducing data completeness. In contrast, the proposed method, by incorporating offset features and semantic segmentation, is better suited to capture the actual geometric characteristics of the tunnel wall. The comparison of tunnel point clouds before and after filtering using the proposed method is shown in Figure 12. This improves filtering stability and provides a more robust foundation for subsequent tunnel point cloud analysis and applications.

### 3.2. Comparison of Segment Segmentation Results

#### 3.2.1. Bolt Hole Results

**Figure 13:**
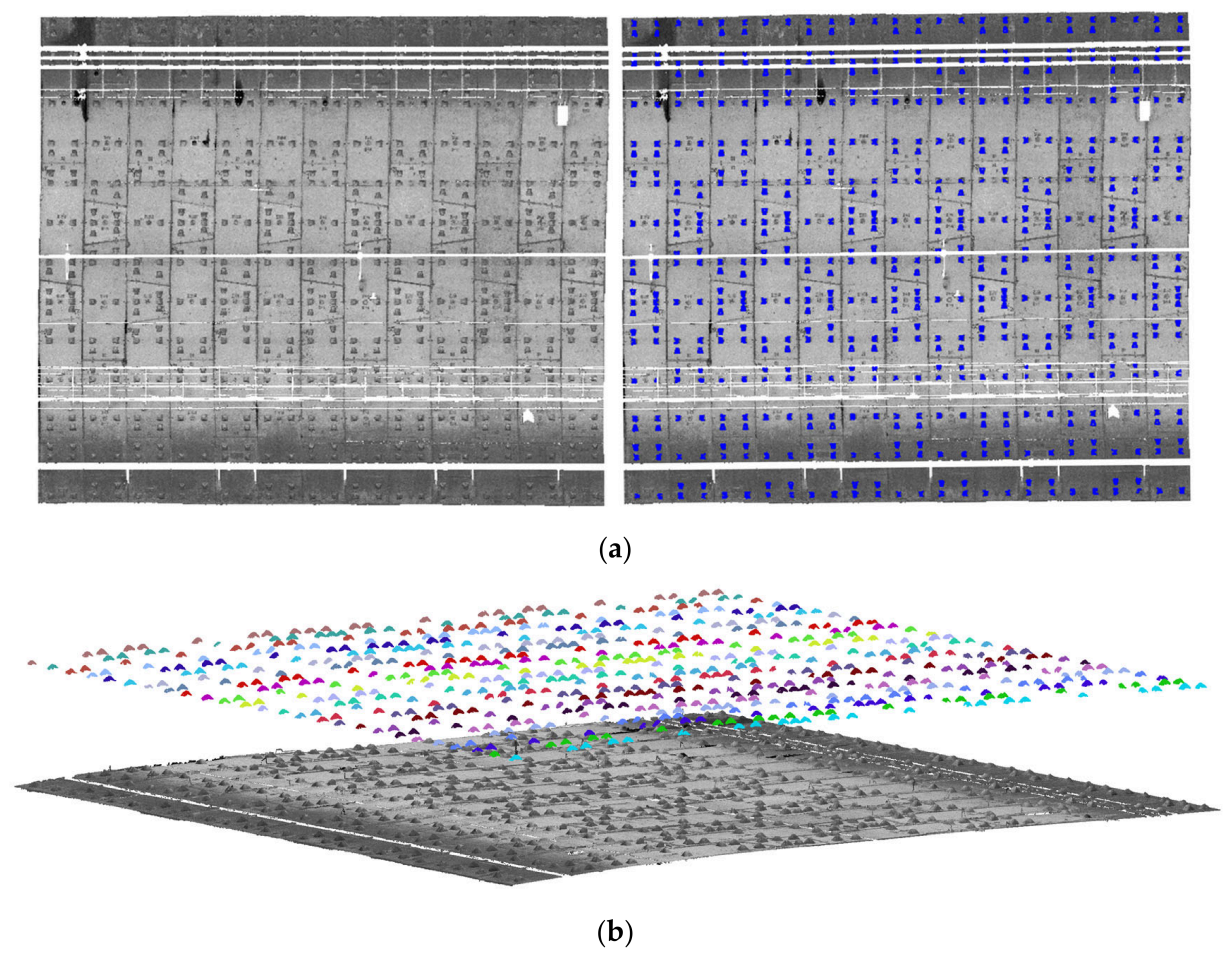
Bolt hole extraction results of this method. (**a**) Comparison of bolt hole extraction results; (**b**) distribution of extracted bolt hole point cloud.

As shown in Figure 13, the proposed algorithm successfully extracts all unobstructed bolt holes in the point cloud data of Ring 13 without introducing any additional noise points. Only one bolt hole was not identified due to occlusion caused by a mileage marker. These results demonstrate that, under conditions where bolt holes are neither blocked nor filled, the proposed bolt hole extraction algorithm achieves excellent performance, accurately distinguishing bolt hole features with high precision and robustness.

#### 3.2.2. Comparison of Segmentation Results

(1) Horizontal Seam Positioning Results

**Figure 14:**
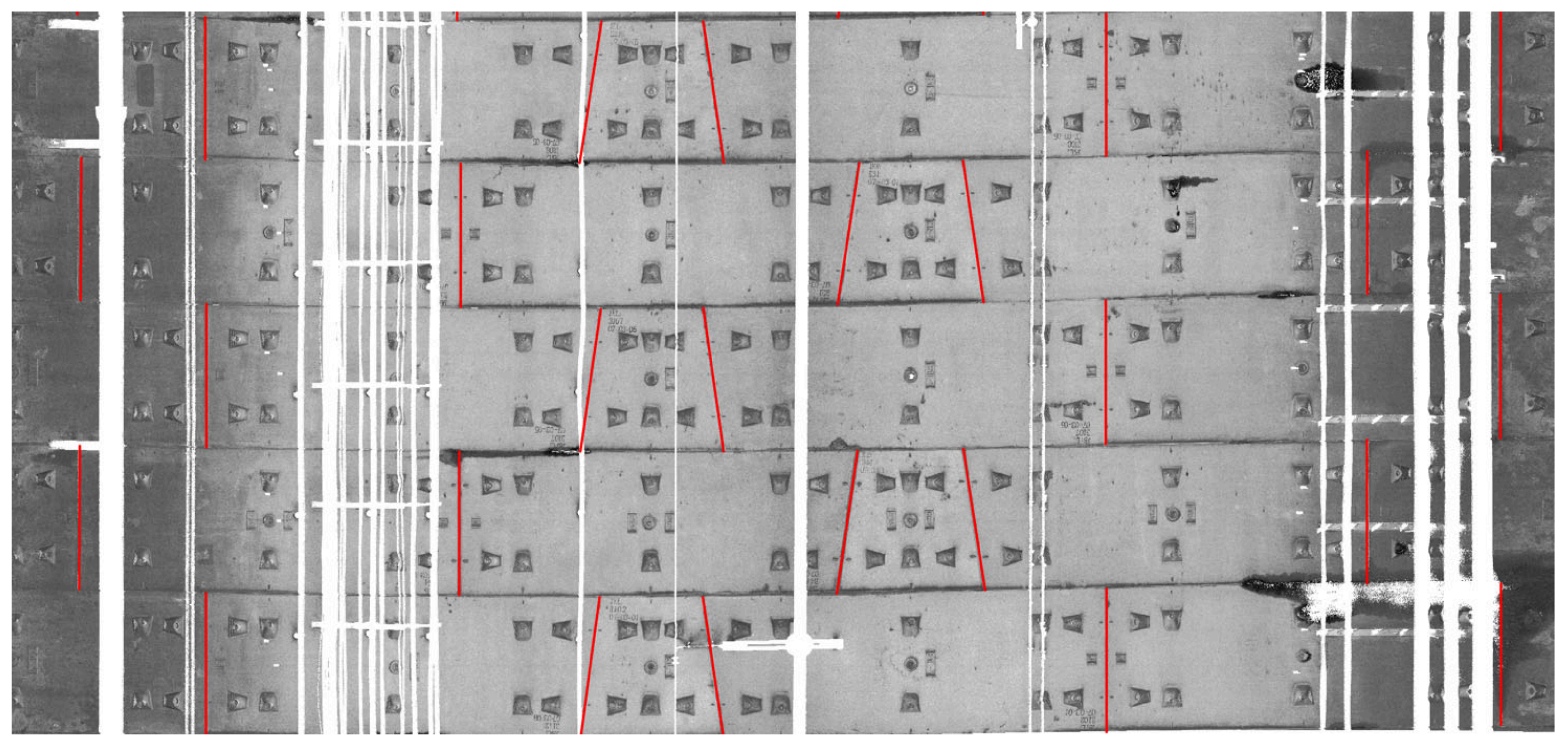
Horizontal seam positioning results.

Figure 14 illustrates the positioning results of the horizontal seams. To evaluate the accuracy of the proposed horizontal seam positioning method, the manually annotated horizontal seam positions are used as the ground truth. The positioning error is calculated by comparing the results of the proposed method with the ground truth values, thereby quantitatively assessing the accuracy and reliability of the algorithm.

As shown in Table 2, the positioning results of the horizontal seams obtained using the method proposed in this paper exhibit minimal deviation from the manually annotated ground truth, with an average error of only 1.6 mm. This demonstrates that the proposed algorithm achieves high accuracy in horizontal seam positioning and is capable of meeting the accuracy requirements for intra-ring dislocation analysis.

(2) Longitudinal seam positioning results

**Figure 15:**
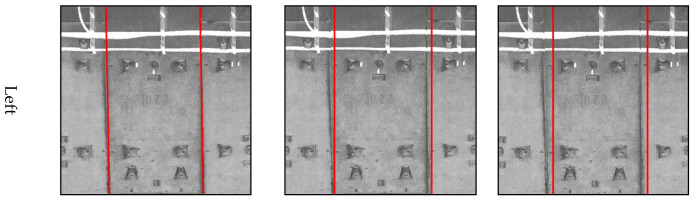

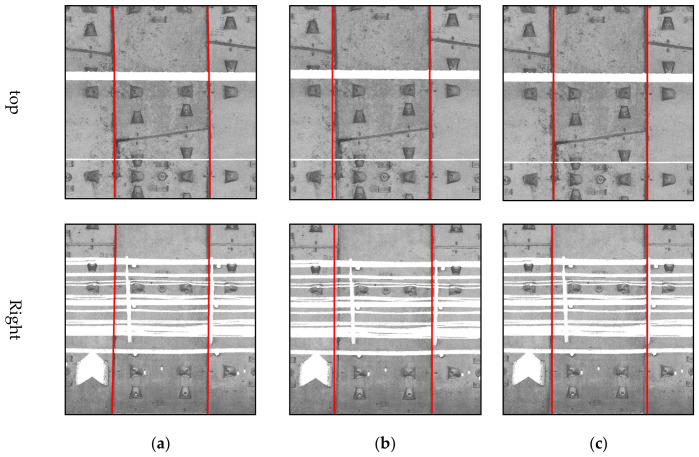
Longitudinal seam positioning results. (**a**) Proposed method; (**b**) Bolt-Center method; (**c**) grayscale thresholding.

In the longitudinal seam positioning experiment, this paper compares the proposed algorithm with two existing methods: the Bolt-Center method [21] and the image grayscale threshold method [33]. The longitudinal seam positioning results obtained by the proposed method and these two baseline approaches are illustrated in Figure 15.

**Figure 16:**
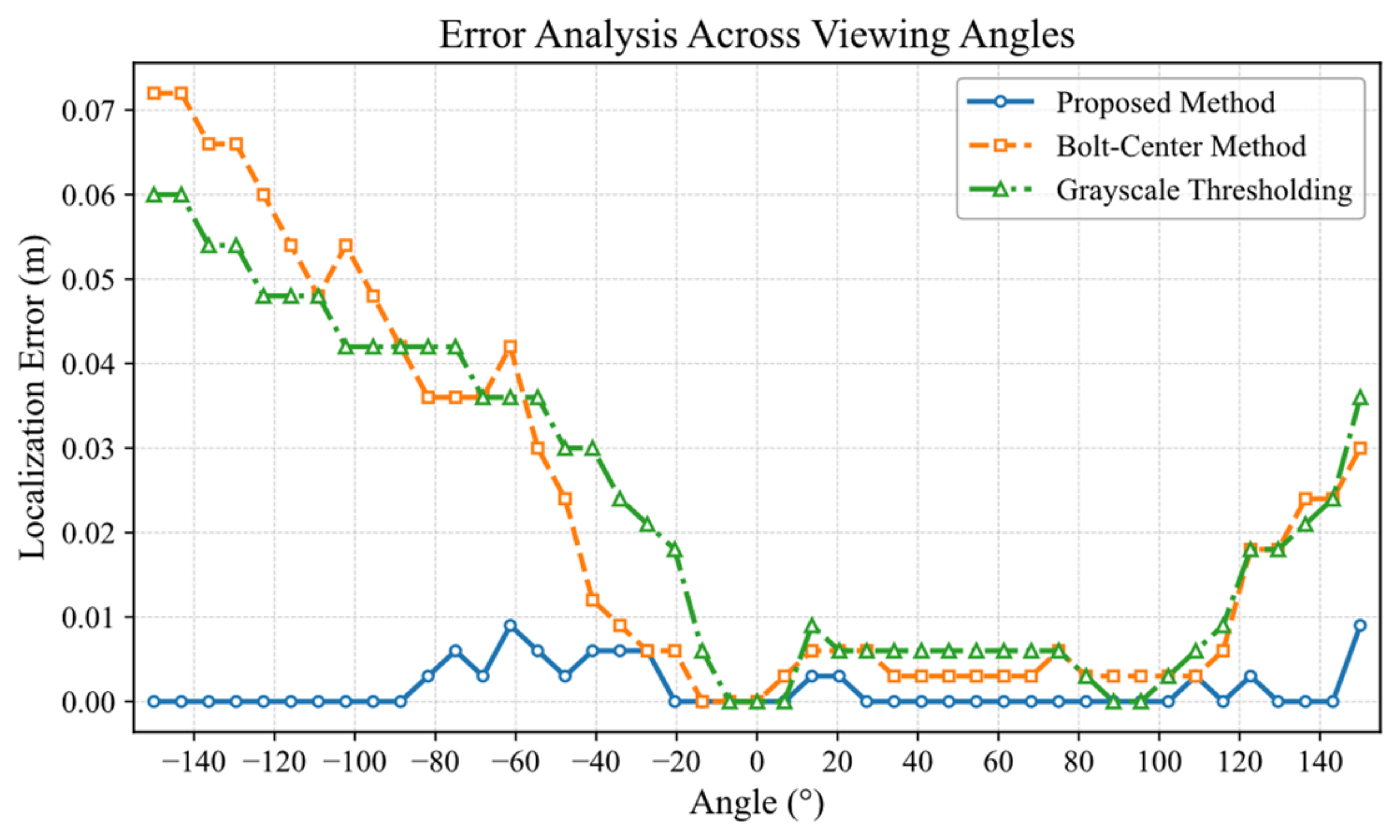
Comparison of longitudinal seam positioning error distribution curves among three methods.

As can be seen intuitively from Figure 15, the longitudinal seam positioning results obtained using the method proposed in this paper exhibit higher accuracy compared to those of the other two methods. To quantitatively assess the performance of each approach, this study calculates the positioning errors at fixed angular intervals along the tunnel and plots the corresponding error distribution curves, as shown in Figure 16. Additionally, the root mean square error (RMSE) of the sampled points is employed as a comprehensive evaluation metric to measure the overall accuracy of the positioning results of each algorithm. The RMSE comparison results of different algorithms are presented in Table 3.

**Figure 17:**
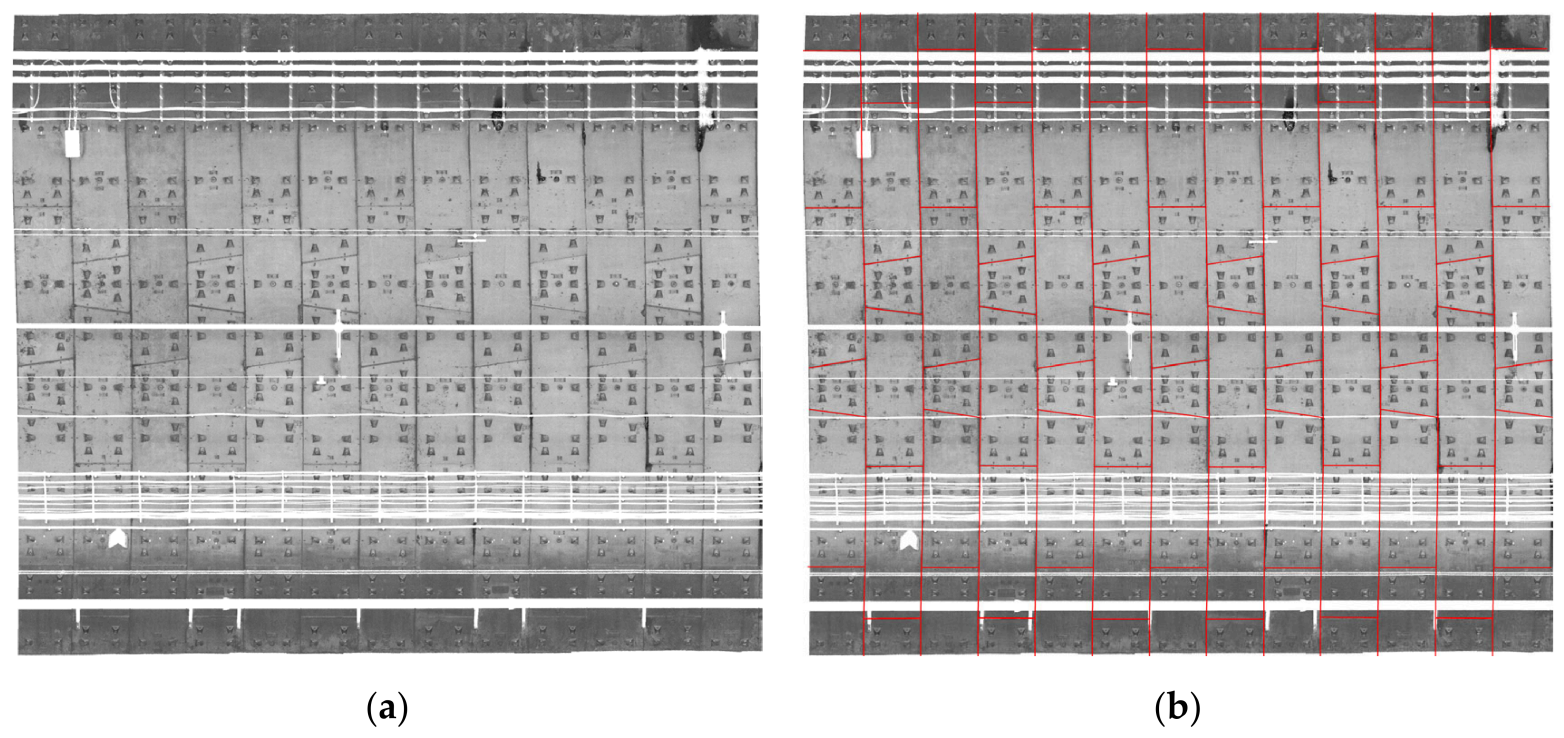
Results of locating the horizontal and longitudinal seams using the proposed method. (**a**) Grayscale image; (**b**) horizontal and longitudinal seam positioning results.

**Figure 18:**
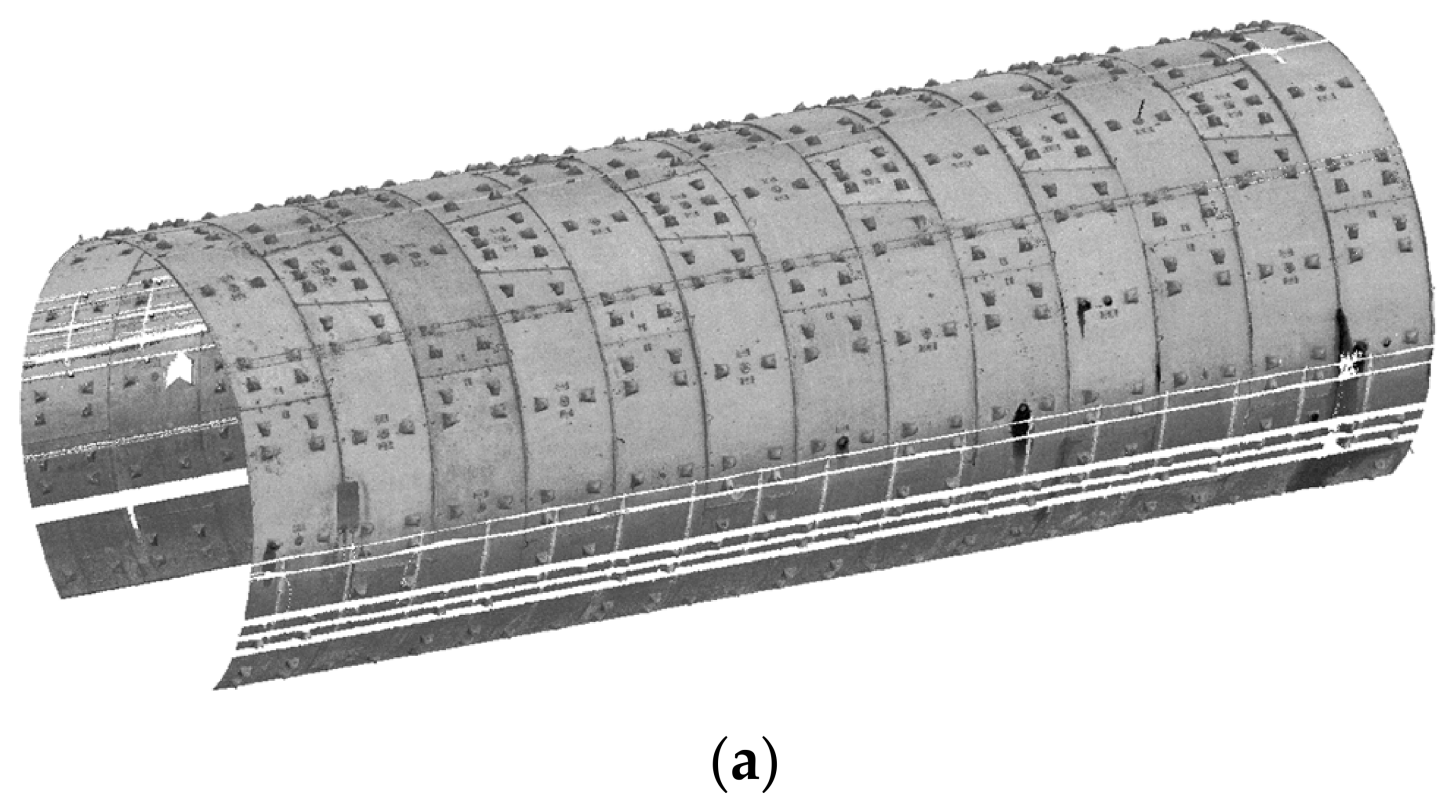

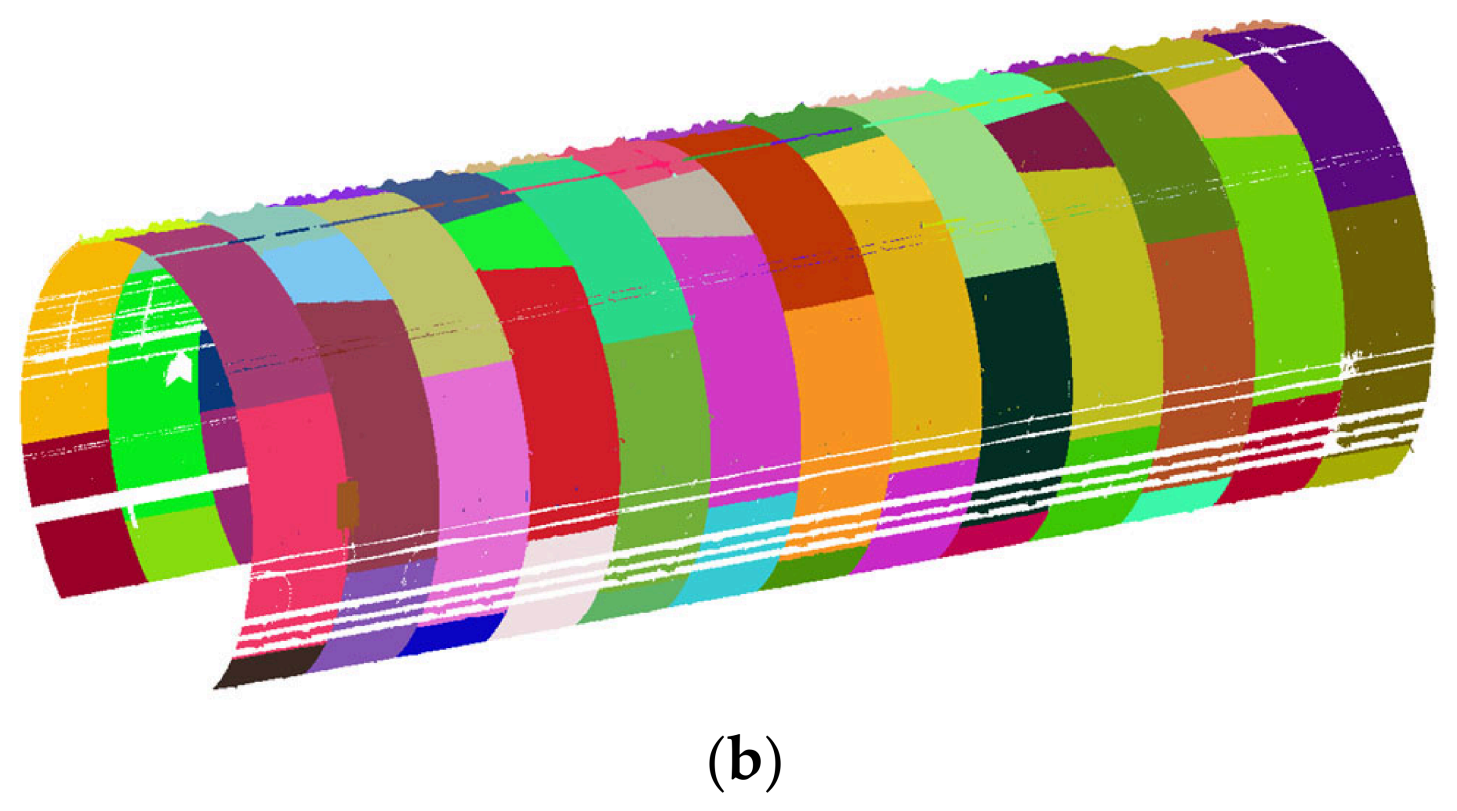
Segment segmentation results. (**a**) Original tunnel point cloud; (**b**) segmented tunnel segment point clouds.

By comparing the experimental data, it can be observed that the positioning error of the proposed method is consistently smaller across all angles, with the overall error significantly lower than that of the other two methods. While the other two methods show higher positioning accuracy at the top of the tunnel, they exhibit lower accuracy along the sides of the tunnel curve. This can be attributed to the fact that the longitudinal seam in the tunnel is not perfectly straight, but instead exhibits certain deformations and offsets. The bolt hole features at the tunnel’s top are relatively clear, and the grayscale values are more distinct, allowing the positioning algorithms to identify the seam with higher precision. However, at the sides of the tunnel, occlusions and stains become more prevalent, leading to less accurate positioning results. As a result, traditional methods demonstrate limited robustness in complex environments. In contrast, the proposed method operates from the segment unit level, allowing it to adapt more effectively to such complexities and thereby improving overall seam positioning accuracy. The results of horizontal and longitudinal seam positioning using the proposed method are shown in Figure 17. Ultimately, based on the horizontal and longitudinal seam positioning results, accurate segmentation of the tunnel segments is achieved. The segment segmentation results are presented in Figure 18.

### 3.3. Deformation Analysis Results

Based on the segment segmentation results, this study investigates both inter-ring and intra-ring dislocations in a subway tunnel using MLS point cloud data. Because independent surveying measurements were unavailable, manually interpreted reference values derived from the same MLS dataset were used as the evaluation reference. Accordingly, the reported errors quantify the consistency between the proposed automatic method and manual interpretation rather than the absolute measurement accuracy of the tunnel geometry. The primary objective of these experiments is to verify that the proposed method can automatically reproduce manual inspection results with high consistency, thereby substantially reducing manual effort while maintaining comparable reliability. In addition, comparative experiments demonstrate that the proposed method achieves higher detection accuracy than existing automatic approaches. In the following experiments, the manually interpreted reference values are regarded as the ground truth for evaluating the performance of the proposed method. The experimental results are presented below.

#### 3.3.1. Inter-Ring Dislocation

**Figure 19:**
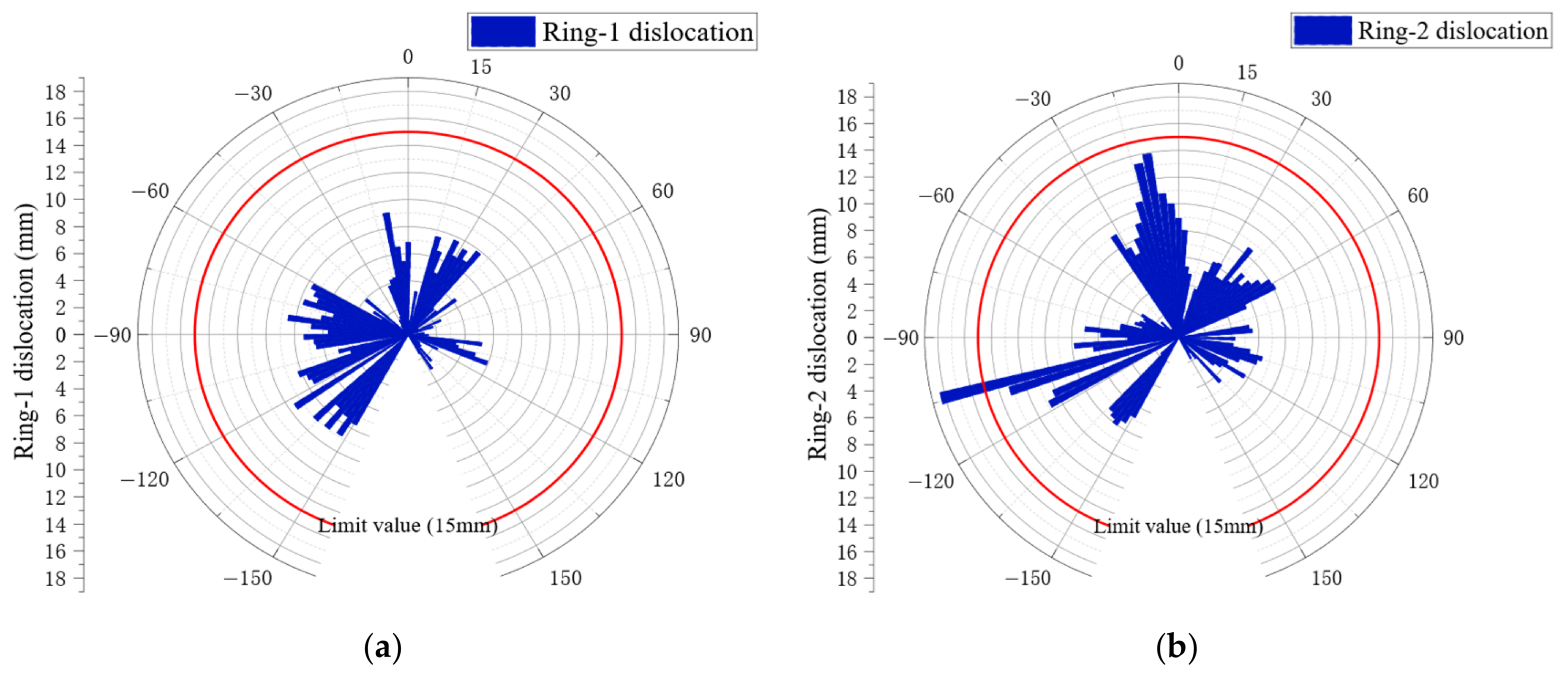

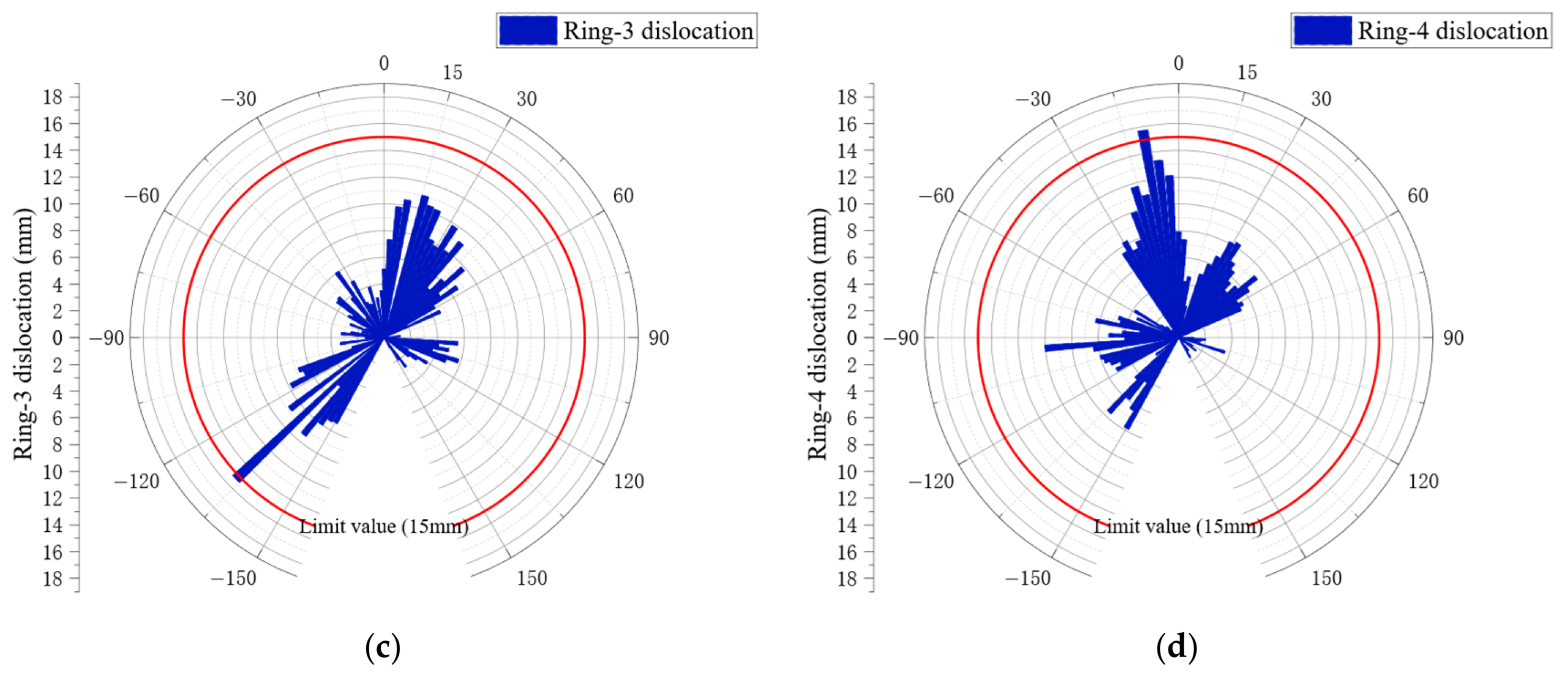
Distribution of inter-ring dislocation angles (with the tunnel vertex as 0° and clockwise as positive angles): (**a**) Ring-1 dislocation; (**b**) Ring-2 dislocation; (**c**) Ring-3 dislocation; (**d**) Ring-4 dislocation.

**Figure 20:**
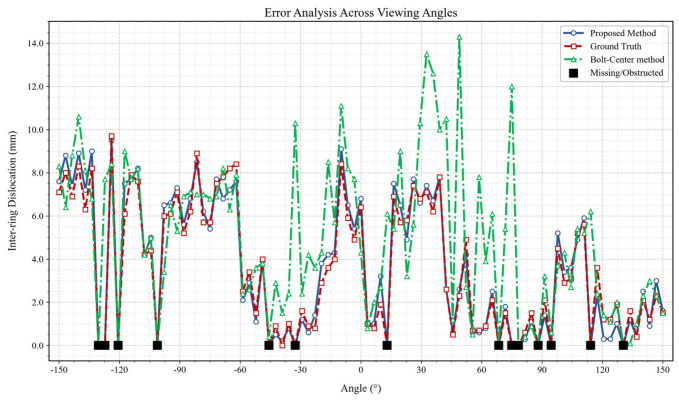
Comparison of inter-ring dislocation results.

**Figure 21:**
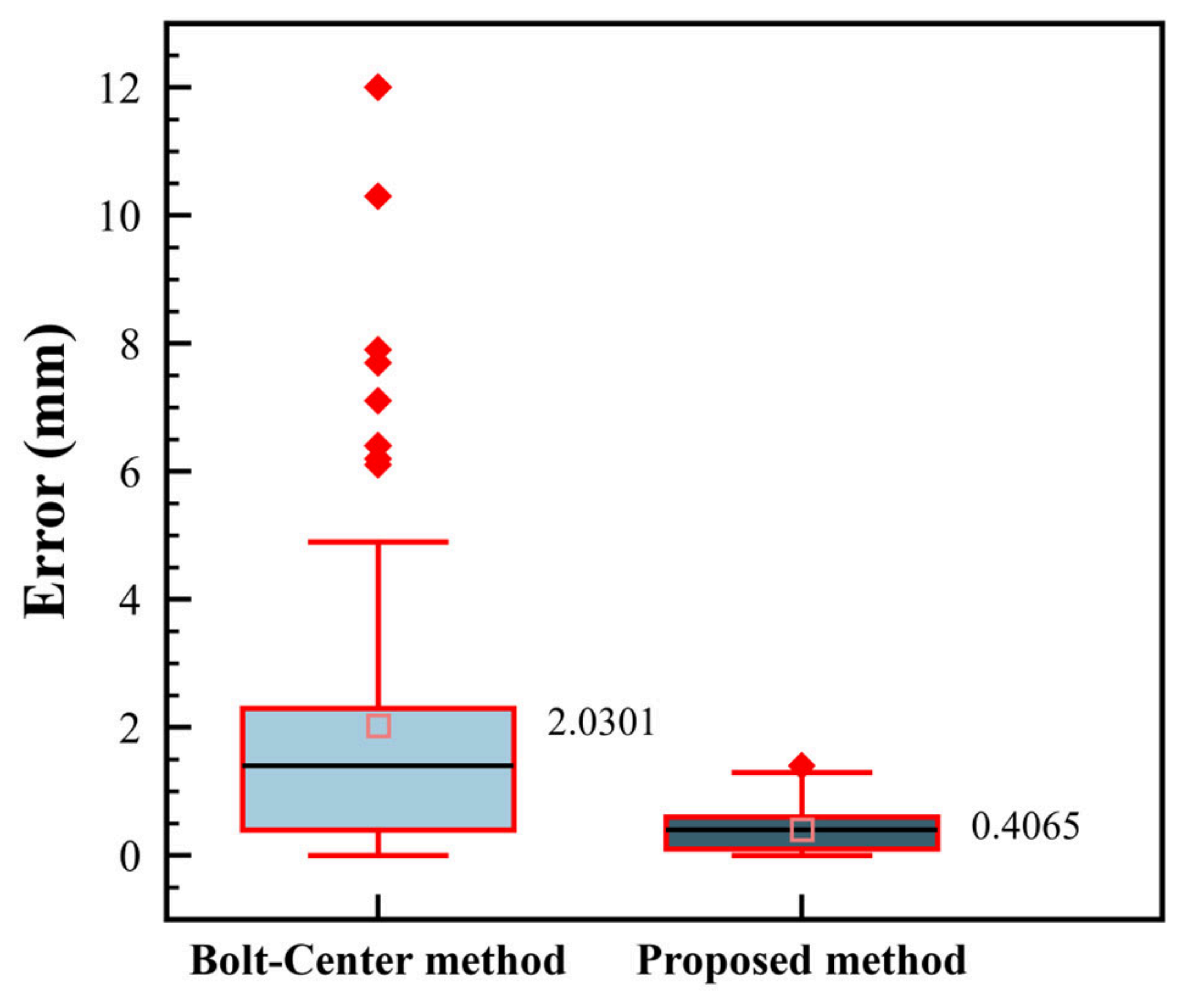
Error box plot of inter-ring dislocation results.

As shown in Figure 19, the second and third rings exhibit noticeable dislocation at different angles, surpassing the 15 mm limit stipulated by the specifications. To verify the measurement accuracy of the proposed algorithm, the inter-ring dislocation results obtained by the proposed algorithm are compared with the manually measured reference values and those obtained using the Bolt-Center method [21]. The comparison of the inter-ring dislocation results and the corresponding error box plots are shown in Figure 20 and Figure 21.

As shown in Figure 20, the inter-ring dislocation results calculated by the proposed algorithm and the Bolt-Center method are close to the manually measured reference values within the angle ranges of −150° to −60° and 90° to 150°, indicating that both algorithms can accurately measure inter-ring dislocation in these ranges. However, between 30° and 70°, the dislocation error of the traditional algorithm increases significantly. This is primarily due to errors in the longitudinal seam positioning within this angle range, which leads to large deviations in the dislocation calculation, further highlighting the limitations of the traditional algorithm. Additionally, at certain angle positions, such as 75° and 114°, the dense distribution of tunnel pipelines leads to inaccurate dislocation calculation results in these areas. The traditional algorithm struggles to effectively distinguish between the tunnel wall and pipeline point clouds, often resulting in false detections, thus reducing its reliability. In contrast, the algorithm proposed in this paper shows a high level of consistency with the true value across the entire angle range, with an overall error controlled within 2mm, demonstrating superior accuracy and stability. Furthermore, the validity verification mechanism for dislocation points in this paper helps avoid erroneous dislocation calculations, improving detection reliability and preventing false detections.

From Figure 21, it is evident that the error distribution of the inter-ring dislocation calculated by the algorithm in this paper is uniform, with the overall error kept within 2 mm. This helps avoid the generation of abnormal data, significantly improving both accuracy and stability.

#### 3.3.2. Intra-Ring Dislocation

**Figure 22:**
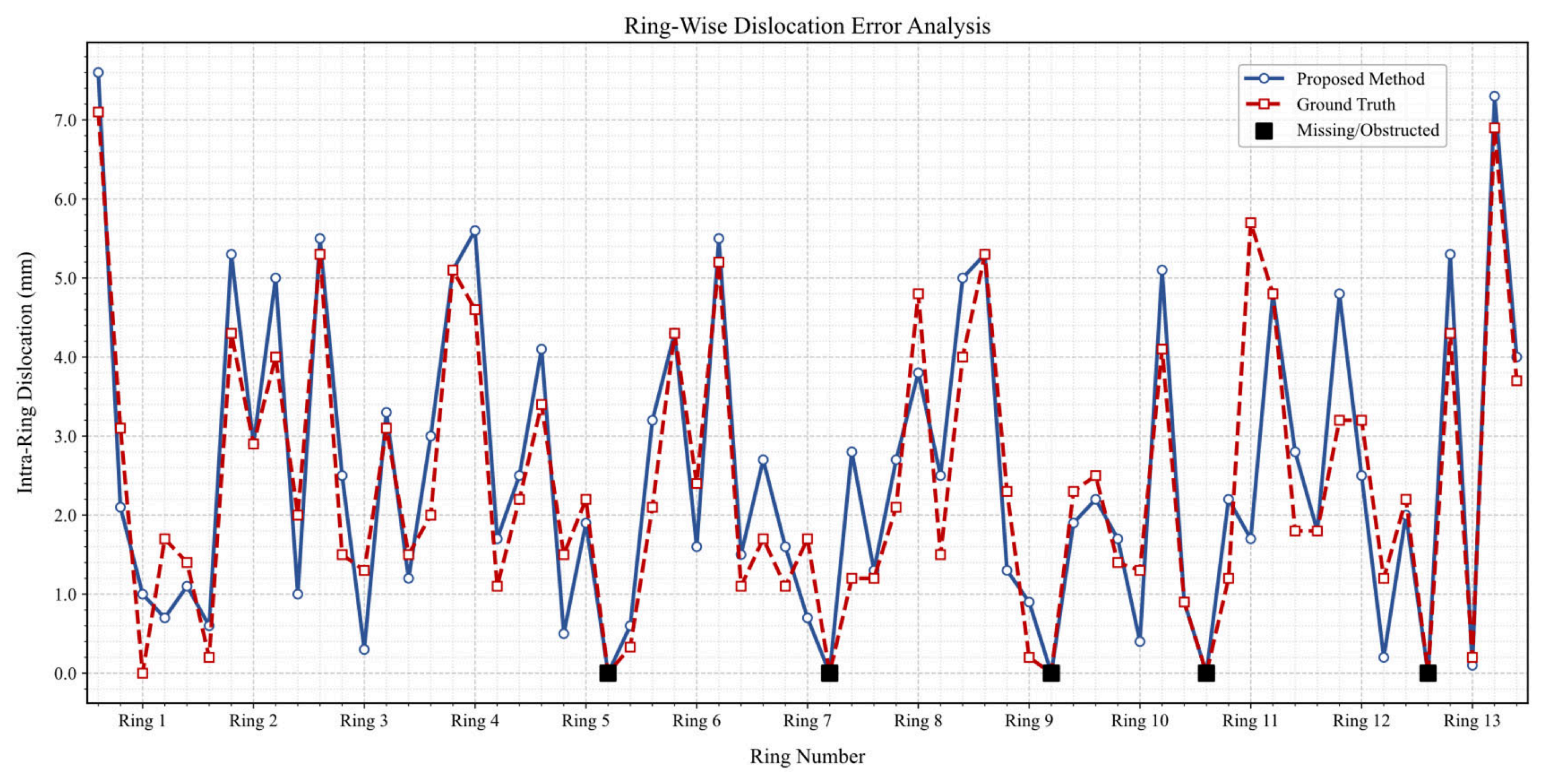
Comparison between the detection result of dislocation within the ring and the true value.

From the analysis of the intra-ring dislocation, it is evident that the proposed algorithm demonstrates high accuracy and stability in measuring the dislocation within the ring. The results of partial intra-ring dislocation measurements are presented in Table 4. The comparison between the detected intra-ring dislocation results and the true values is shown in Figure 22. This performance is also attributed to the precise positioning of the horizontal seam.

Overall, the proposed method effectively overcomes the influence of inaccurate seam localization and noise points in MLS point clouds on dislocation analysis. By combining robust point cloud filtering, accurate segment seam localization, and automated dislocation calculation, the proposed approach significantly reduces manual intervention and improves the automation and efficiency of tunnel segment deformation detection.

## 4. Discussion

The experimental results verify the effectiveness of the proposed framework for shield tunnel point cloud processing. For tunnel wall point cloud filtering, the proposed method outperforms ellipse fitting and cylinder fitting, as shown in Figure 11 and Table 1, with accuracy improvements of 8.7% and 5.6%, respectively. This indicates that real tunnel linings cannot be fully described by ideal ellipse or cylinder models because of construction errors, geological conditions, and deformation. By combining offset features with semantic segmentation, the proposed method can better preserve valid tunnel wall points while removing noise.

For seam positioning, the horizontal seam results are evaluated using manually annotated seam positions as the ground truth, as shown in Figure 14. The proposed method achieves an average positioning error of 1.6 mm. For longitudinal seam positioning, the method is compared with the Bolt-Center method [21] and the image grayscale threshold method [33], as shown in Figure 15. The proposed method shows smaller errors under different angles, especially in areas affected by occlusion, stains, and local joint deformation. This suggests that segment-level structural analysis can improve the robustness of seam positioning in complex tunnel environments.

The deformation analysis further confirms the reliability of the proposed method. As shown in Figure 20 and Figure 21, the traditional method [22] produces larger errors in some angle ranges because longitudinal seam positioning errors and pipeline interference are propagated into the dislocation calculation. In contrast, the proposed method reduces false detections through validity verification of dislocation points, and the overall dislocation error is controlled within 2 mm. The intra-ring dislocation results also show good stability, mainly benefiting from the high accuracy of horizontal seam positioning. Accurate seam localization effectively avoids deformation analysis errors caused by incorrect seam extraction and noisy point clouds, thereby improving the reliability of dislocation detection.

From a practical perspective, the proposed framework is designed for automated processing of MLS point clouds and can be integrated into existing tunnel inspection workflows. The method consists of independent modules, including point cloud filtering, seam positioning, and dislocation analysis, making it convenient for deployment and future upgrades. Since the proposed framework mainly relies on local geometric features and deep-learning-based semantic segmentation, it can process large-scale tunnel point clouds with good scalability. The modular design also facilitates the replacement or optimization of individual components without affecting the overall workflow, providing flexibility for engineering applications.

Nevertheless, several limitations remain. First, the proposed offset feature is constructed based on the geometric prior of circular shield tunnels. Therefore, the current method is mainly applicable to circular shield tunnels and cannot be directly applied to rectangular, horseshoe-shaped, or composite tunnel cross-sections. For other tunnel types, the circular reference model could be replaced by corresponding design-section models or piecewise parametric models, while the offset feature can be redefined as the signed distance from a point to the local reference surface. In addition, seam templates and matching strategies should be adapted according to different segment layouts and joint topologies. Second, this study focuses on geometric deformation detection from MLS point clouds and does not explicitly consider structural mechanical characteristics, such as segment joint stiffness, bolt connection conditions, surrounding ground pressure, water pressure, or longitudinal constraints. Integrating the detected dislocation with structural mechanical models and multi-temporal monitoring data would improve the interpretation of deformation mechanisms and enable condition assessment and risk prediction, which will be an important direction for future research.

## 5. Conclusions

This study proposes an automated shield tunnel point cloud analysis framework integrating geometric feature perception, semantic segmentation, seam localization, and dislocation deformation detection. The main conclusions are summarized as follows:

(1) The proposed point cloud filtering method effectively improves the extraction accuracy of tunnel lining points compared with conventional ellipse fitting and cylinder fitting methods. The accuracy is increased by 8.7% and 5.6%, respectively. By combining offset geometric features with semantic segmentation, the proposed method can better distinguish valid tunnel lining points from noise while preserving the structural characteristics of tunnel surfaces.
(2) The proposed seam localization method achieves accurate detection of both horizontal and longitudinal segment joints. The average positioning error of horizontal seams is 1.6 mm. Compared with the Bolt-Center method [21] and the image grayscale threshold method [33], the proposed method provides higher positioning accuracy and stronger robustness under complex conditions, including occlusion, surface stains, and local joint deformation.
(3) The proposed deformation analysis method enables accurate detection of both inter-ring and intra-ring dislocation. The overall dislocation error is controlled within 2 mm. The reliable deformation measurement benefits from accurate seam localization and validity verification of dislocation points, which effectively reduces false detections caused by seam positioning errors and noisy point clouds.
(4) The proposed framework provides an automated and modular solution for shield tunnel point cloud processing, integrating point cloud filtering, seam localization, and deformation analysis into a unified workflow.

## 6. Patents

The method proposed in this paper, entitled Point Cloud Filtering Method for Subway Shield Tunnels Integrating Offset Features and Semantic Segmentation, has been granted a Chinese invention patent. The patent number is ZL 2024 1 0951437.7, and the authorization announcement number is CN 118587398 B. The patentee is China University of Mining and Technology.

## Figures and Tables

**Table 1:** Comparison of three filtering methods.

Filtering Algorithm	Accuracy (%)	Precision (%)	Recall (%)
Ellipse fitting filtering	91.17	63.63	78.31
Cylindrical fitting filtering	93.84	75.67	79.04
Proposed method	99.07	99.20	93.79

**Table 2:** Comparison of the horizontal seam locations of the proposed method and the true value.

Ring Number	Horizontal Seam Position	True Positions of the Seams (m)	Algorithm Positioning Position (m)	Algorithm Error (mm)
1	A2P-A1P	−6.034	−6.031	−3
	A1P-BP	−2.716	−2.714	−2
	BP-KP	0.602	0.601	1
	KP-CP	1.641	1.638	3
	CP-A3P	4.874	4.871	3
2	A1P-BP	−4.912	−4.907	−5
	BP-KP	−1.589	−1.588	−1
	KP-CP	−0.534	−0.534	0
	CP-A3P	2.74	2.737	3
	A3P-A2P	5.936	5.936	0
3	A2P-A1P	−6.031	−6.028	−3
	A1P-BP	−2.71	−2.708	−2
	BP-KP	0.599	0.599	0
	KP-CP	1.626	1.624	2
	CP-A3P	4.866	4.866	0
4	A2P-A1P	−6.032	−6.031	−1
	A1P-BP	−2.711	−2.711	0
	BP-KP	0.601	0.601	0
	KP-CP	1.626	1.627	−1
	CP-A3P	4.874	4.876	−2
Average error (mm)				1.6

**Table 3:** RMSE comparison of different algorithms.

	Evaluation Indicators	RMSE (m)
Methods		
Proposed method		0.0030
Bolt-Center method		0.0317
Grayscale thresholding		0.0296

**Table 4:** Results of partial intra-ring dislocation (“/” indicates invalid dislocation measurements).

Ring Number	Dislocation in Different Rings (mm)					
	A2P-A1P	A1P-BP	BP-KP	KP-CP	CP-A3P	A3P-A2P
1	−7.6	2.1	1	−0.7	−1.1	/
2	/	0.6	5.3	2.9	−5	1
3	−5.5	2.5	−0.3	−3.3	1.2	/
4	/	−3	5.1	5.6	−1.7	2.5
5	−4.1	0.5	−1.9	/	−0.6	/
6	/	−3.2	4.3	1.6	/	1.5
7	−2.7	1.6	−0.7	/	2.8	/
8	/	−1.3	2.7	3.8	−2.5	5
9	−5.3	1.3	−0.9	/	1.9	/
10	/	−2.2	1.7	−0.4	−5.1	0.9

## Data Availability

The data used in this study are not publicly available due to confidentiality and data protection requirements of the subway operating company involved in this project. The datasets were accessed under a restricted agreement and cannot be redistributed or shared publicly.

## References

[1] Ministry of Transport of the People’s Republic of China China’s Urban Rail Transit Operating Mileage Exceeds 11,710 Kilometers. https://www.mot.gov.cn/xinwen/jiaotongyaowen/202601/t20260129_4199263.html.

[2] Yang Y., Zhang Y., Tan X. (2021). Review on Vibration-Based Structural Health Monitoring Techniques and Technical Codes. Symmetry.

[3] Sainoki A., Tabata S., Mitri H.S., Fukuda D., Kodama J.-I. (2017). Time-Dependent Tunnel Deformations in Homogeneous and Heterogeneous Weak Rock Formations. Comput. Geotech..

[4] Janda T., Šejnoha M., Šejnoha J. (2018). Applying Bayesian Approach to Predict Deformations during Tunnel Construction. Int. J. Numer. Anal. Methods Geomech..

[5] Paraskevopoulou C., Diederichs M. (2018). Analysis of Time-Dependent Deformation in Tunnels Using the Convergence-Confinement Method. Tunn. Undergr. Space Technol..

[6] Balaguer C., Montero R., Victores J.G., Martínez S., Jardón A. Towards Fully Automated Tunnel Inspection: A Survey and Future Trends. Proceedings of the 31st International Symposium on Automation and Robotics in Construction (ISARC), Sydney, Australia.

[7] Kavvadas M.J. (2005). Monitoring Ground Deformation in Tunnelling: Current Practice in Transportation Tunnels. Eng. Geol..

[8] Chen D., Wang X., Bai J., Lu J., Wu B., Li X., Li Y., Zhang F., Li M. (2025). Advanced Detection Methods for Tunnels and Roadways: A Review. Meas. Sci. Technol..

[9] Wang X., Wang M., Jiang R., Xu J., Li B., Wang X., Yu J., Su P., Liu C., Yang Q. (2024). Structural Deformation Monitoring during Tunnel Construction: A Review. J. Civ. Struct. Health Monit..

[10] Shen N., Wang B., Ma H., Zhao X., Zhou Y., Zhang Z., Xu J. (2023). A Review of Terrestrial Laser Scanning (TLS)-Based Technologies for Deformation Monitoring in Engineering. Measurement.

[11] Ding N., Zhou Y., Li D., Zeng K. (2024). Real-Time Deformation Monitoring of Large Diameter Shield Tunnel Based on Multi-Sensor Data Fusion Technique. Measurement.

[12] Cui H., Ren X., Mao Q., Hu Q., Wang W. (2019). Shield Subway Tunnel Deformation Detection Based on Mobile Laser Scanning. Autom. Constr..

[13] Wu H.N., Shen S.L., Liao S.M., Yin Z. (2015). Longitudinal structural modelling of shield tunnels considering shearing dislocation between segmental rings. Tunn. Undergr. Space Technol..

[14] Zhang D.M., Liu J., Huang Z.K., Yang G.H., Jiang Y., Jia K. (2022). Waterproof performance of tunnel segmental joints under different deformation conditions. Tunn. Undergr. Space Technol..

[15] Jin Y., Ding W., Yan Z., Soga K., Li Z. (2017). Experimental investigation of the nonlinear behavior of segmental joints in a water-conveyance tunnel. Tunn. Undergr. Space Technol..

[16] Yao L., Sun H., Xu Z., Wu H. (2022). Application of Mobile Laser Measurement New Technologies in Rail Transit.

[17] Du L., Zhong R., Sun H., Liu Y., Wu Q. (2019). Cross-Section Positioning Based on a Dynamic MLS Tunnel Monitoring System. Photogramm. Rec..

[18] Sun H., Xu Z., Yao L., Zhong R., Du L., Wu H. (2020). Tunnel Monitoring and Measuring System Using Mobile Laser Scanning: Design and Deployment. Remote Sens..

[19] Yi C., Lu D., Xie Q., Xu J., Wang J. (2020). Tunnel Deformation Inspection via Global Spatial Axis Extraction from 3D Point Cloud. Sensors.

[20] Chen L., Ge K., Wang T. (2020). Central Axis Extraction Method of Shield Tunnel Based on Ring Seam Point Cloud. J. Hohai Univ. Nat. Sci..

[21] Lu J., Li W., Yan Z., Bao Y., Wu X., Ni S. (2022). Circumferential Seam Detection and Analytics of Segment Misplacement Between Rings of Subway Shield Tunnels Based on Featured Point Cloud of Bolt Holes. Bull. Surv. Mapp..

[22] Yu A., Mei W., Han M. (2021). Deep Learning-Based Method of Longitudinal Dislocation Detection for Metro Shield Tunnel Segment. Tunn. Undergr. Space Technol..

[23] Lu D.N. (2021). Research on Tunnel Structural Deformation Detection Technology Based on Mobile 3D Laser Scanning. Master’s Thesis.

[24] Cheng S., Sang Z.L., Wu Y., Zheng J.J., Lu J.J. (2022). Tunnel Segment Dislocation Detection Based on Mobile 3D Laser Scanning. Geomat. Spat. Inf. Technol..

[25] Zhang Y., Ren X., Zhang J., Ma Z. (2024). A Method for Deformation Detection and Reconstruction of Shield Tunnel Based on Point Cloud. J. Constr. Eng. Manag..

[26] Li X., Wu B. (2021). Denoising Method of Tunnel Point Cloud Data Based on Ellipse Fitting. J. Railw. Sci. Eng..

[27] Xu X., Yang H., Neumann I. (2018). Time-Efficient Filtering Method for Three-Dimensional Point Cloud Data of Tunnel Structures. Adv. Mech. Eng..

[28] Chen K., Liu H., Yan L. (2021). A Tunnel Point Cloud Filtering Algorithm by RANSAC Multi-Model Fitting. Surv. Mapp. Geogr. Inf..

[29] Zhu N., Jia Y., Luo L. (2016). Tunnel Point Cloud Filtering Method Based on Elliptic Cylindrical Model. Int. Arch. Photogramm. Remote Sens. Spat. Inf. Sci..

[30] Shi B., Yang M., Liu J., Han B., Zhao K. (2023). Rail Transit Shield Tunnel Deformation Detection Method Based on Cloth Simulation Filtering with Point Cloud Cylindrical Projection. Tunn. Undergr. Space Technol..

[31] Choudhury P., Tumblin J. The Trilateral Filter for High Contrast Images and Meshes. Proceedings of the ACM SIGGRAPH 2005 Courses.

[32] Qi C.R., Yi L., Su H., Guibas L.J. (2017). PointNet++: Deep Hierarchical Feature Learning on Point Sets in a Metric Space. Adv. Neural Inf. Process. Syst..

[33] Liu X., Chen Y., Liu X. (2021). Laser Scanning-Based Rapid Detection of Deformation of Shield Tunnel Section. J. Traffic Transp. Eng..

